# Temporal Trends in Preterm Birth Associated With Hypertensive Disorders of Pregnancy in Victoria, Australia: A Population‐Based Interrupted Time‐Series Study

**DOI:** 10.5694/mja2.70267

**Published:** 2026-08-24

**Authors:** Melvin Marzan, Heng Jiang, Daniel Lorber Rolnik, Joanne M. Said, Lisa Hui

**Affiliations:** ^1^ Department of Obstetrics, Gynecology and Newborn Health University of Melbourne Heidelberg Victoria Australia; ^2^ Reproductive Epidemiology Murdoch Children's Research Institute Parkville Victoria Australia; ^3^ Mercy Perinatal Mercy Hospital for Women Heidelberg Victoria Australia; ^4^ National Centre for Register‐Based Research Aarhus University Aarhus Denmark; ^5^ School of Psychology and Public Health La Trobe University Melbourne Victoria Australia; ^6^ Centre for Health Equity, School of Population and Global Health University of Melbourne Melbourne Victoria Australia; ^7^ Department of Obstetrics and Gynaecology Monash University Clayton Victoria Australia; ^8^ Department of Obstetrics and Gynaecology Monash Health Clayton Victoria Australia; ^9^ Department of Maternal Fetal Medicine Joan Kirner Women's and Children's, Sunshine Hospital St Albans Victoria Australia; ^10^ Department of Obstetrics and Gynaecology Northern Health Epping Victoria Australia

**Keywords:** high‐risk pregnancy, hypertension, perinatal, preterm birth, preterm labour, women's health

## Abstract

**Objectives:**

Hypertensive disorders of pregnancy (HDP) are a leading reason for medically indicated preterm birth (PTB), yet their contribution to population‐level PTB trends is poorly understood. We examined temporal trends in PTB overall and HDP‐associated PTB in Victoria over an 11‐year period spanning the COVID‐19 pandemic, and distinguished changes in HDP prevalence from changes in preterm delivery among affected pregnancies.

**Study Type:**

Population‐based interrupted time‐series study using seasonal autoregressive integrated moving average (SARIMA) models, with July 2018 (launch of a national preterm birth prevention program) pre‐specified as a temporal reference point.

**Setting:**

All hospitals in Victoria, Australia.

**Participants:**

779,325 singleton births at ≥ 20 weeks' gestation between 1 January 2012 and 31 March 2022.

**Main Outcome Measures:**

Monthly rates (per 1000 singleton births) of overall PTB, HDP and HDP‐associated PTB. Decomposition analyses separated changes in HDP prevalence from changes in preterm delivery among affected pregnancies.

**Results:**

Overall PTB was lower after July 2018 (5.8%–5.6%), while HDP prevalence increased (6.4%–7.0%). HDP‐associated PTB increased before mid‐2018 (+0.75 per 1000 births per month; 95% confidence interval [CI], 0.50–1.00) but declined thereafter (−1.12 per 1000 births per month; 95% CI, −1.88 to −0.36). This reversal was driven predominantly by reduced iatrogenic PTB with HDP; spontaneous PTB with HDP showed no significant change. Despite rising HDP prevalence, decomposition analyses estimated 89 fewer HDP‐associated PTB cases than expected under pre‐2018 patterns, reflecting a decline in the proportion of HDP pregnancies delivered preterm. PTB without HDP showed no significant temporal change.

**Conclusion:**

PTB among HDP‐complicated pregnancies declined despite increasing HDP prevalence, consistent with a shift towards later gestational age at delivery rather than reduced disease incidence. These findings may reflect improvements in care and demonstrate that reductions in iatrogenic PTB are achievable even as major risk factors become more prevalent.

## Plain Language Summary

**The Known:** Preterm birth (birth before 37 weeks of pregnancy) is a major cause of infant illness and death. High blood pressure during pregnancy is a common and serious condition and the leading reason for medically indicated, planned preterm birth.

**The New:** Using data from all Victorian singleton births between 2012 and 2022, we found that high blood pressure during pregnancy became more common, but preterm birth among affected pregnancies declined. This was mainly due to fewer medically recommended preterm births, while preterm birth after spontaneous preterm labour showed little change.

**The Implications:** Although high blood pressure during pregnancy is increasing, more affected pregnancies are now reaching full‐term gestation, suggesting improved outcomes for mothers and newborns. This reduction in preterm birth with hypertension may reflect changes in the severity of the hypertensive disorders, changes in clinical care or both.

## 1 Background

Preterm birth (PTB) remains a leading cause of neonatal mortality worldwide, responsible for more than one million deaths each year and substantial long‐term consequences for children, families and health systems [1]. In Australia, PTB affects about 8% of pregnancies and has proven resistant to sustained population‐level reduction despite decades of research and clinical innovation [2].

Hypertensive disorders of pregnancy (HDP), including chronic hypertension, gestational hypertension and pre‐eclampsia/eclampsia, represent the leading cause for medically indicated PTB. HDP are also associated with an increased risk of spontaneous PTB through placental insufficiency, decidual vasculopathy and premature rupture of membranes [3, 4]. Although the prevalence of HDP has increased over time, driven in part by rising maternal age and cardiometabolic risk, the contribution of HDP to total PTB trends and how this contribution may be changing, remain poorly understood [5]. Importantly, a PTB associated with HDP is potentially modifiable. Risk stratification can identify pregnancies that benefit from established preventive strategies, including low‐dose aspirin prophylaxis in individuals at elevated risk of pre‐eclampsia [6, 7], intensified antenatal surveillance and clinically judicious timing of delivery [8]. Although spontaneous preterm labour is multifactorial, evidence‐based approaches such as universal cervical length screening and subsequent treatment with progesterone and/or cerclage in high‐risk individuals also demonstrate preventive potential at a population level [9].

A prior study of PTB and HDP in Victoria reported stable prevalences to 2017 [10], but this study period did not capture recent national and jurisdictional initiatives to reduce preventable PTB in Australia [11, 12]. The national PTB prevention program has prioritised preventing non‐medically indicated early delivery, led to wider dissemination of evidence supporting expectant management when clinically appropriate and targeted prevention strategies for selected high‐risk groups [2]. The recent evaluation of this national PTB prevention program reported reductions in PTBs following program implementation [11]. However, that analysis did not examine condition‐specific drivers of PTB or distinguish between changes in disease prevalence and changes in obstetric management, limiting insight into the mechanisms underlying observed improvements.

Using whole‐of‐population data from Victoria, Australia, we conducted a population‐based time‐series study to examine temporal trends in total PTB and PTB associated with HDP between 2012 and 2022. We aimed to: (1) characterise changes in the temporal trends of PTB with HDP before and after a pre‐specified period of national practice transition; (2) distinguish changes in HDP prevalence from changes in the likelihood of preterm delivery among affected pregnancies; and (3) explore whether trends differed across hospital, geographical, socio‐economic and gestational age subgroups. By focusing on HDP‐specific PTB, this study seeks to provide insight into the component pathways underlying recent population‐level trends and to inform strategies for further reducing preventable PTB.

## 2 Methods

We conducted a population‐based observational time‐series analysis of all singleton hospital births in Victoria between 1 January 2012 and 31 March 2022. This period captures the impact of the COVID‐19 pandemic, specifically the periods of intensive lockdown in Melbourne between 2020 and 2021. The study followed the Strengthening the Reporting of Observational Studies in Epidemiology (STROBE) guidelines [13] (Table S1). Ethical approval was obtained from the Royal Children's Hospital Human Research Ethics Committee (QA/82339/RCHM‐2021).

### 2.1 Data Source and Study Population

Data were obtained from the Victorian Admitted Episodes Dataset (VAED), which captures all public and private hospital admissions in Victoria (Department of Health Victoria) and includes de‐identified demographic, diagnostic, procedural and administrative information. We included all hospital admissions for singleton births from 20 weeks' gestation to women aged 15 years or older. We excluded multifetal pregnancies and abortions. Monthly denominators comprised all eligible singleton births, and all results were expressed as the rate per 1000 births. Although time‐series models used data up to March 2022, decomposition analysis was restricted to 2012–2021 to avoid seasonal bias from partial‐year data. The VAED is a mandatory administrative dataset with high completeness for the variables used in this study. Only socio‐economic status had missing variables (*n* = 2938, 0.38%). We calculated monthly numerators and denominators from complete records and did not impute missing data.

### 2.2 Definitions of Exposures and Outcomes

HDP was identified using the *International Statistical Classification of Diseases and Related Health Problems*, Tenth Revision, Australian Modification (ICD‐10‐AM) [14] diagnostic codes for chronic hypertension (O10, O100–O104, O109), gestational hypertension (O13), pre‐eclampsia and its subtypes (O11, O140–O142, O149) and eclampsia (O150–O152, O159), together with unspecified maternal hypertension (O16).

PTB was defined as birth at < 37 completed weeks' gestation. Subtypes of PTB were categorised as spontaneous (O601 ‘Preterm spontaneous labour with preterm delivery’) or iatrogenic (O603 ‘Preterm delivery without spontaneous labour’).

### 2.3 Outcomes

The primary outcome was the temporal trend in monthly rates of births with both PTB and HDP.

Secondary outcomes included temporal trends in monthly rates of total PTB, PTB subtypes (iatrogenic and spontaneous), and total and term HDP.

Exploratory analyses examined whether trends in PTB with HDP differed by pre‐specified subgroups. These included preterm gestational age group (20–33^+6^ or 34–36^+6^ weeks: based on ICD‐10‐AM coding), hospital type (public or private), geographical remoteness (metropolitan or regional) and socio‐economic status (Index of Relative Socio‐economic Advantage and Disadvantage quintiles). A composite indicator of severe adverse pregnancy outcomes, defined as maternal intensive care unit (ICU) admission or stillbirth, was examined descriptively for pregnancies with both PTB and HDP.

### 2.4 Temporal Reference Point

We pre‐specified July 2018 as the temporal reference point for segmented time‐series analyses. This period coincided with the introduction of evolving clinical guidance related to PTB prevention in Australia, including the official launch of the national PTB prevention program [2]. The national PTB prevention program promoted evidence‐based strategies including discouraging non‐medically indicated PTBs and implementing universal transabdominal second trimester cervical length screening, building on earlier successful implementation in Western Australia [15] (Box S1). Other key developments in PTB and HDP over the past decade include the 2014 Society of Obstetric Medicine of Australia and New Zealand (SOMANZ) guidelines for the diagnosis and management of HDP [8], and the publication of the ASPRE (Aspirin for Evidence‐Based Pre‐eclampsia Prevention) trial in 2017, which demonstrated the effectiveness of aspirin for preventing preterm pre‐eclampsia among high‐risk individuals identified by a first‐trimester screening program [7]. Following these results from the ASPRE trial, there were changes in recommendations by multiple guidelines in Australia and worldwide. Rather than representing a single discrete intervention, the reference point of 2018 was used to examine whether population‐level trends in PTB and HDP changed during this period of practice transition.

Joinpoint regression was undertaken to objectively identify inflection points [16] in observed total PTB trends (further details in the Supporting Information). This analysis detected a significant change in slope in December 2017, preceding the pre‐specified July 2018 reference point by 6 months. This finding is consistent with gradual uptake of emerging evidence and clinical practice changes before formal national implementation given the absence of pandemic‐era shifts, and supports the use of July 2018 as a reference period for examining changes in trends over time. December 2017 was not used as the primary reference point to avoid data‐driven specification of the segmented model.

### 2.5 Statistical Analysis

We addressed three complementary objectives within the overall aim to examine trends in PTB and HDP. Analyses were conducted across two periods: Period 1 (January 2012–June 2018) and Period 2 (July 2018–March 2022), separated by the pre‐specified temporal reference point described above.

First, we compared descriptive characteristics and outcome prevalences between Periods 1 and 2.

Second, to characterise temporal trends and assess changes in slope over time, we modelled monthly outcome rates using Seasonal Autoregressive Integrated Moving Average (SARIMA) models. SARIMA was chosen because it jointly accommodates trend, seasonal and short‐lag autocorrelation within a single parsimonious model suited to segmented regression at a pre‐specified inflection point [17], which standard Poisson or negative‐binomial generalised linear models cannot natively handle without violating the independence‐of‐residuals assumption [17]. We estimated population‐level monthly rates rather than individual‐level associations and therefore did not incorporate individual‐level covariate adjustment; differences in population composition over time were examined descriptively (Table 1) and through stratified analyses. The SARIMA model accounts for autocorrelation and seasonal variation commonly observed in population health time‐series data [19, 20]. Monthly outcomes were modelled on the absolute rate scale (cases per 1000 singleton births) rather than as proportions; observed rates lay well within the interval where a logit transformation is about linear, and we therefore retained the untransformed scale to preserve direct interpretability of coefficients. This approach builds on our group's previous interrupted time‐series analysis of Victorian abortion data [21].

**TABLE 1:** Sociodemographic characteristics of singleton births by study period.

Sociodemographic characteristics	Period 1 January 2012–June 2018 (*n* = 449,373)	Period 2 July 2018–March 2022 (*n* = 329,952)
	*n* (%)	*n* (%)
Geographical remoteness		
Metropolitan	343,951 (76.5)	255,205 (77.3)
Regional	105,422 (23.5)	74,747 (22.7)
SEIFA‐IRSAD quintiles		
1 (most disadvantaged)	65,779 (14.6)	44,714 (13.6)
2	72,606 (16.2)	54,463 (16.5)
3	98,798 (22.0)	73,395 (22.2)
4	103,492 (23.0)	75,698 (22.9)
5 (most advantaged)	107,797 (24.0)	79,645 (24.1)
Missing	901 (0.2)	2037 (0.6)
Hospital classification		
Private	111,963 (24.9)	73,837 (22.4)
Public	337,410 (75.1)	256,115 (77.6)

*Note:* Study periods reflect a pre‐specified temporal reference point corresponding to a period of national clinical practice transition. This table describes the characteristics of all singleton births and is intended for descriptive purposes only; temporal trends were formally assessed using time‐series models.

Abbreviation: SEIFA‐IRSAD = Socio‐Economic Indexes for Areas, Index of Relative Socio‐economic Advantage and Disadvantage [18].

We used the forecast package in R [22], with model preference determined by ranking the values of the model‐specific Akaike Information Criterion. Temporal reference effects were introduced as indicator variables capturing both immediate level changes and post‐temporal reference slope changes. Regression coefficients with 95% confidence intervals (CIs) were reported, representing absolute monthly changes in outcome rates. Stratified SARIMA analyses were conducted by hospital type, remoteness, socio‐economic status and gestational age band. A seasonal component (period s = 12 months) was retained because automatic model selection consistently identified non‐zero seasonal orders, and ignoring modest but present seasonality can inflate residual autocorrelation and bias trend inference (Figure S1). Third, to assess whether changes in PTB with HDP reflected shifts in HDP prevalence or changes in the likelihood of preterm delivery among HDP‐affected pregnancies, we performed decomposition analyses [23]. Expected numbers of PTB with HDP after July 2018 were estimated by applying the January 2012–December 2017 PTB rate among women with HDP to the observed HDP prevalence and total births, generating an expected scenario assuming no change in clinical management and continuous trends. The difference between expected and observed counts represented the net divergence from Period 1 trends (Table S2).

All analyses were conducted using R (version 4.3.1) [24]. Statistical significance was assessed at a two‐sided *α* level of 0.05.

## 3 Results

### 3.1 Study Population and Baseline Characteristics

A total of 779,325 singleton hospital births were included: 449,373 (57.7%) in Period 1 (January 2012 to June 2018) and 329,952 (42.3%) during Period 2 (July 2018 to March 2022) (Table 1). Median maternal age was higher in Period 2 (32, interquartile range [IQR] 28–35 years old) compared with Period 1 (31, [IQR 27–35] years old).

### 3.2 Total Prevalence of PTB and HDP

The prevalence of total PTB was slightly lower in Period 2 than Period 1 (5.6% vs. 5.8%, respectively). In contrast, the prevalence of HDP was higher in Period 2 than Period 1 (7.0% vs. 6.4%, respectively) (Table 2).

**TABLE 2:** Prevalence of preterm and term births and hypertensive disorders of pregnancy by study period, Victoria, Australia, 1 January 2012 to 31 March 2022.

	Period 1 January 2012–June 2018 (*n* = 449,373)	Period 2 July 2018–March 2022 (*n* = 329,952)
	*n* (%)	*n* (%)
Total preterm births	25,912 (5.8)	18,588 (5.6)
Total term births	423,461 (94.2)	311,364 (94.4)
Total births with HDP	28,564 (6.4)	23,157 (7.0)
Preterm^a^	3968 (13.9)	3128 (13.5)
Term^a^	24,596 (86.1)	20,029 (86.5)
Severe adverse outcomes in births with PTB and HDP^b^, ^c^	95 (2.4)	58 (1.9)

*Note:* HDP was defined using ICD‐10‐AM codes O13, O10, O16, O100–O104 and O109, representing pre‐existing hypertension complicating pregnancy, labour or the puerperium; O140 (mild‐to‐moderate pre‐eclampsia), O141 (severe pre‐eclampsia), O149 (unspecified pre‐eclampsia), O11 (pre‐eclampsia superimposed on chronic hypertension), O142 (HELLP syndrome) and O150–O152, O159 (eclampsia at various time points).

Abbreviations: HDP = hypertensive disorders of pregnancy, HELLP = haemolysis, elevated liver enzymes, low platelets, ICD‐10‐AM = International Statistical Classification of Diseases and Related Health Problems, Tenth Revision, Australian Modification, ICU = intensive care unit, NICU = neonatal intensive care unit, Preterm birth = birth before 37 completed weeks' gestation, PTB = preterm birth, SCN = special care nursery, term birth = birth at 37 completed weeks' gestation or later, VAED = Victorian Admitted Episodes Dataset.

^a^ Denominators are all births with HDP.

^b^ Denominators are preterm births with HDP.

^c^ Severe adverse pregnancy outcomes were defined as maternal ICU admission or stillbirth. Neonatal outcomes (e.g., NICU/SCN admission, neonatal death) were not included because they are either mechanically confounded by gestational age at delivery or subject to ascertainment limitations within a single VAED birth episode.

Severe adverse outcomes (maternal ICU admission or stillbirth) among pregnancies complicated by PTB and HDP were uncommon but lower in Period 2 than Period 1 (1.9 per 100 PTBs in Period 2 vs. 2.4 per 100 PTBs in Period 1).

### 3.3 Primary Outcome: Temporal Trends in PTBs With HDP

**FIGURE 1:**
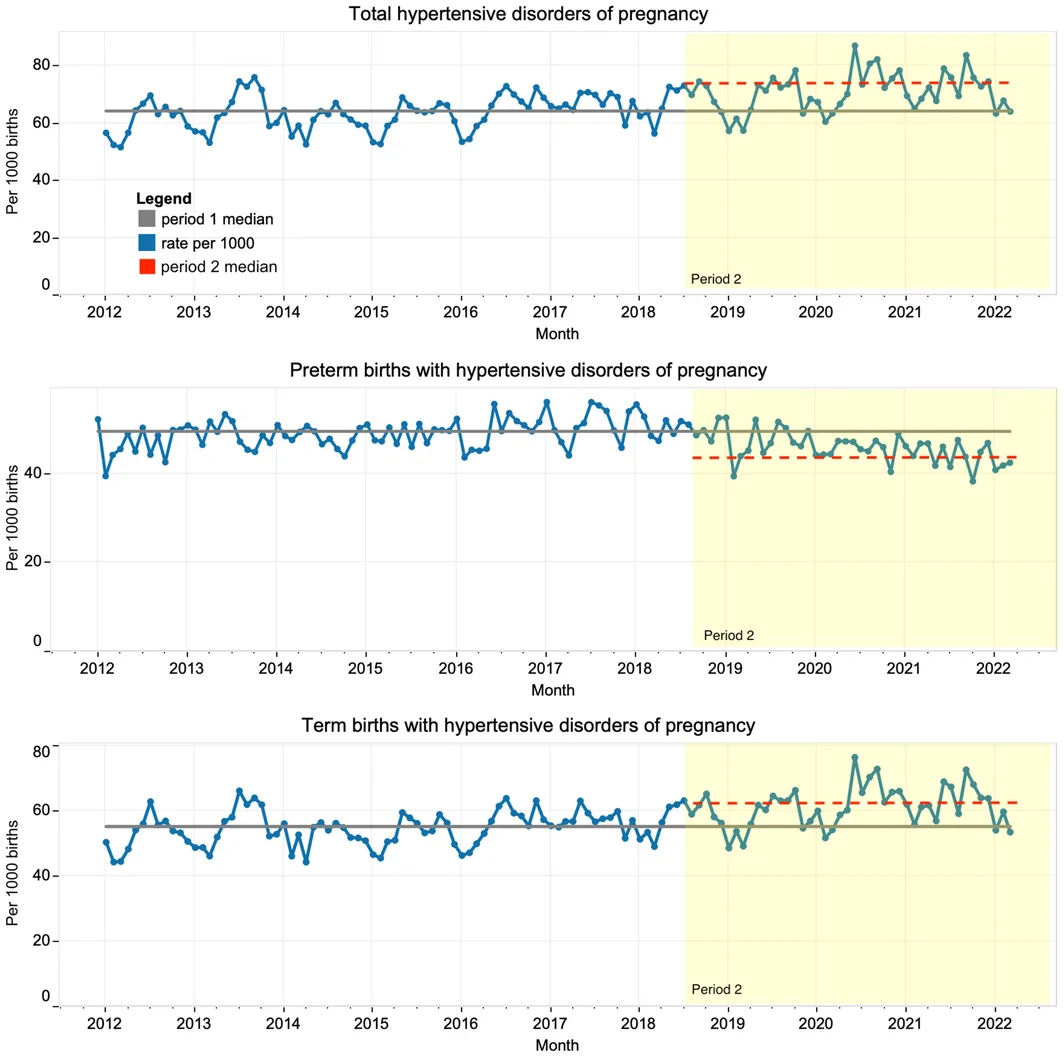
Trends in monthly rates of hypertensive disorders of pregnancy. Preterm birth = birth before 37 completed weeks' gestation; term birth = birth at 37 completed weeks' gestation or later.

The temporal trend in PTBs with HDP showed a clear change in trajectory over the study period (Figure 1, Table 3, Table S3). SARIMA models demonstrated a significant increasing trend before mid‐2018 (+0.75 cases per 1000 births per month; 95% CI, 0.50–1.00), followed by a significant reversal in trend following mid‐2018 (−1.12 per 1000 births per month; 95% CI, −1.88 to −0.36) (Table 3). This resulted in the Period 2 slope of −0.37 per 1000 births per month.

**TABLE 3:** Seasonal autoregressive integrated moving average (SARIMA) analysis of preterm birth and hypertensive disorders of pregnancy trends by study period.

Primary and secondary outcomes	Period 1 slope (95% CI) January 2012–June 2018	Change in slope (95% CI)	Period 2 slope (95% CI) July 2018–March 2022^a^
PTB with HDP	0.751 (0.503 to 0.999)***	−1.122 (−1.884 to −0.360)**	−0.371 (−0.946 to 0.205)
Iatrogenic PTB with HDP	0.100 (0.008 to 0.191)*	−0.086 (−0.310 to 0.138)	0.014 (−0.148 to 0.175)
Spontaneous PTB with HDP	0.018 (−0.016 to 0.052)	−0.014 (−0.101 to 0.074)	0.004 (−0.057 to 0.065)
Total PTB	0.518 (−0.549 to 1.585)	−0.364 (−2.514 to 1.785)	0.154 (−1.294 to 1.602)
Total iatrogenic PTB	0.341 (−0.031 to 0.713)	−0.420 (−1.203 to 0.363)	−0.079 (−0.622 to 0.465)
Total spontaneous PTB	0.048 (−0.409 to 0.505)	0.102 (−0.605 to 0.809)	0.150 (−0.401 to 0.700)
Total HDP	0.405 (−0.440 to 1.250)	−0.103 (−1.408 to 1.203)	0.302 (−0.685 to 1.290)
Term pregnancies with HDP	0.027 (0.005 to 0.048)*	−0.011 (−0.050 to 0.029)	0.016 (−0.013 to 0.044)
Term pregnancies without HDP	0.120 (0.106 to 0.134)***	−0.177 (−0.244 to −0.110)***	−0.057 (−0.124 to 0.010)
PTB without HDP	0.097 (−0.041 to 0.236)	−0.073 (−0.364 to 0.218)	0.025 (−0.177 to 0.227)
Iatrogenic PTB without HDP	0.220 (−0.085 to 0.526)	−0.365 (−0.934 to 0.204)	−0.145 (−0.541 to 0.251)
Spontaneous PTB without HDP	0.058 (−0.381 to 0.497)	0.076 (−0.608 to 0.759)	0.134 (−0.393 to 0.660)

*Note:* Coefficients represent absolute monthly change in rate per 1000 births | *< 0.05, **< 0.01, ***< 0.001. Estimates represent the absolute monthly change in the rate per 1000 singleton births. 95% confidence intervals are derived from SARIMA model standard errors.

Abbreviations: CI = confidence interval, HDP = hypertensive disorders of pregnancy, Preterm birth = birth before 37 completed weeks' gestation, PTB = preterm birth, term birth = birth at 37 completed weeks' gestation or later.

^a^ Period 1 slope (*β*_t_) represents the baseline trend. The Change in Slope (*β*_ramp1_) represents the shift in trajectory following the intervention. The Period 2 slope is the absolute trend for the second period, calculated as the linear combination of the baseline slope and the change: Period 2 slope = *β*_t_ + *β*_ramp1_.

When examined by PTB subtype, iatrogenic PTB with HDP increased significantly in Period 1 (+0.10 per 1000 per month; 95% CI, 0.01–0.19). Although the trend reversed in direction in Period 2, the change in slope was not significant (−0.09 per 1000 births per month; 95% CI, −0.31–0.14). Spontaneous PTB with HDP showed no significant temporal change in either period (Table 3).

Results were unchanged in sensitivity analyses using December 2017 as the reference point. In a sensitivity analysis restricted to the pre‐lockdown period (excluding April 2020 onwards), the direction of effect for PTB with HDP was preserved (Period 1 slope: 0.42 per 1000 births per month; 95% CI, −0.51–1.35; change in slope: −0.26 per 1000 births per month; 95% CI, −2.43–1.91), although confidence intervals widened and the estimates did not reach statistical significance, reflecting the reduced power from using December 2017 as the reference point (Table S4).

### 3.4 Secondary Outcomes: Temporal Trends in Total PTB, PTB Subtypes and HDP

**FIGURE 2:**
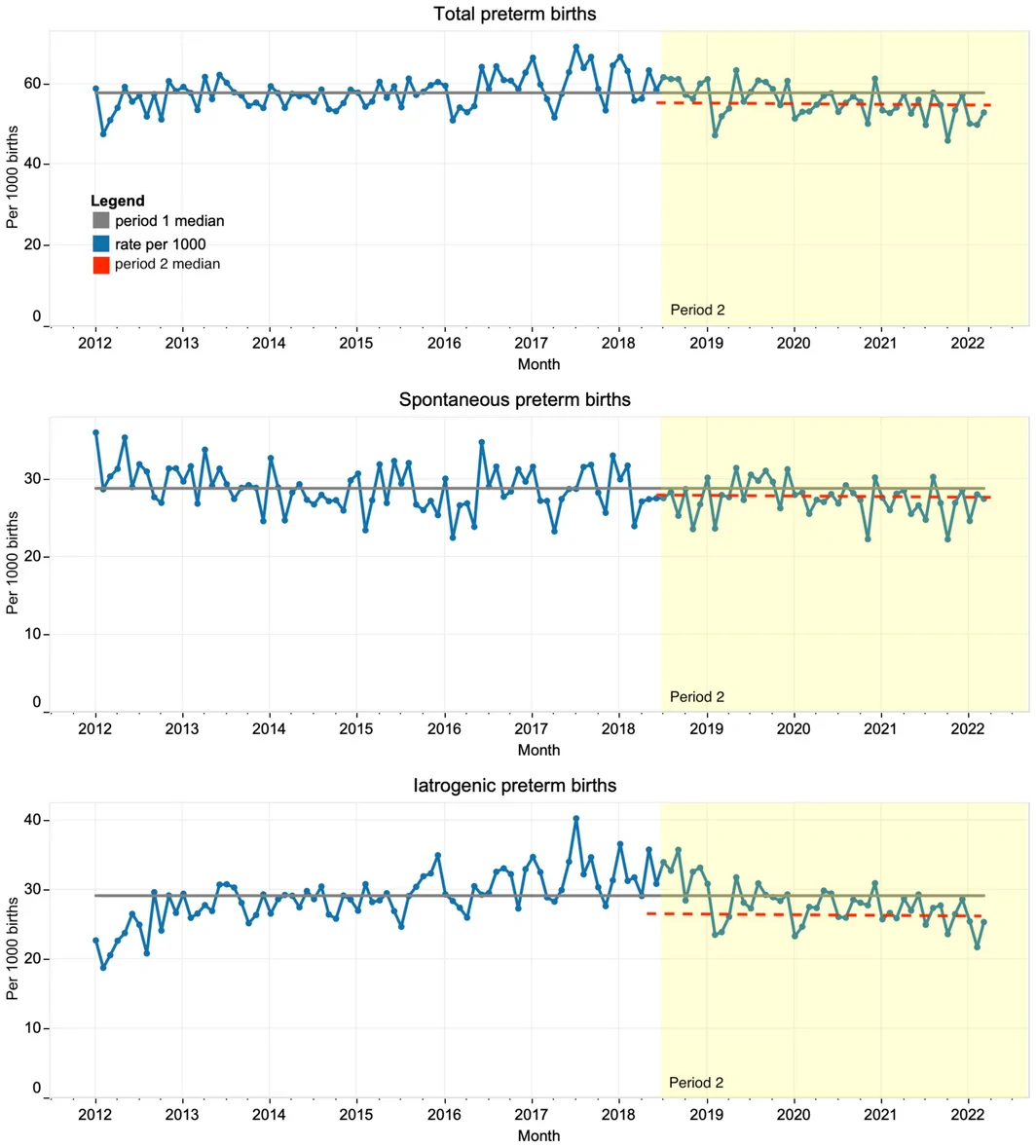
Trends in monthly total preterm births and its subtypes. Preterm birth = birth before 37 completed weeks' gestation; term birth = birth at 37 completed weeks' gestation or later.

Total PTB showed no significant trend in Period 1 (0.52 per 1000 births per month; 95% CI, −0.55 to 1.59) and no statistically significant change in slope following the intervention (−0.36 per 1000 births per month; 95% CI, −2.51 to 1.79) (Figure 2, Table 3). Similarly, total iatrogenic PTB (change in slope: −0.42 per 1000 births per month; 95% CI, −1.20 to 0.36) and spontaneous PTB (change in slope: 0.10 per 1000 births per month; 95% CI, −0.61 to 0.81) demonstrated no significant changes in trajectory across the two periods.

The prevalence of HDP showed an increasing trend in Period 1 (0.41 per 1000 births per month; 95% CI, −0.44 to 1.25), with no significant change in slope following the intervention (−0.10 per 1000 births per month; 95% CI, −1.41 to 1.20), indicating continued increase throughout the study period (Figure 2, Table 3). PTB without HDP, including both iatrogenic and spontaneous subtypes, showed no significant Period 1 trends or changes in slope following the intervention.

### 3.5 Exploratory Subgroup Analyses

Exploratory stratified analyses suggested that trends in iatrogenic PTB with HDP varied across selected subgroups (Table S5 and Figures S2–S5). In regional areas and among more socio‐economically disadvantaged populations, positive trend slopes for iatrogenic PTB with HDP in Period 1 reversed to downward trajectories during Period 2, although these declines did not consistently reach statistical significance. Similar but attenuated patterns were observed in private hospitals and among births at 34–36^+6^ weeks' gestation. Trends for iatrogenic PTB without HDP showed comparable but smaller changes, whereas no consistent subgroup‐specific patterns were observed for spontaneous PTB.

### 3.6 Decomposition of PTB Among Women With HDP

**FIGURE 3:**
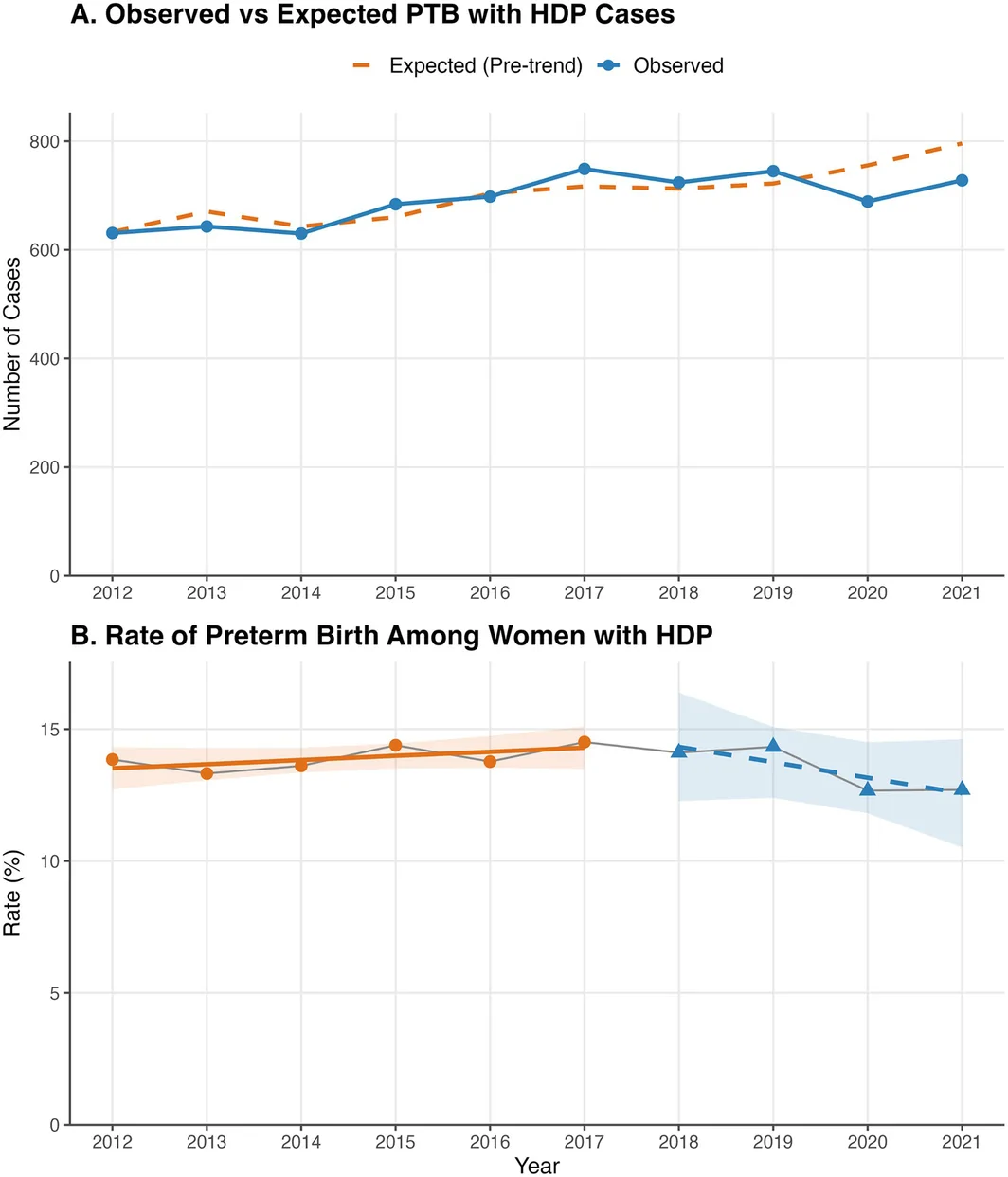
Year‐by‐year decomposition analysis of preterm birth with hypertensive disorders of pregnancy. (A) The observed annual PTB with HDP cases (blue solid line) compared with expected cases; the expected (Pre‐trend, orange dashed line) shows expected cases if the 2012–2017 trend had continued after the temporal reference point in 2018 (shaded area indicates Period 2). (B) The rate of PTB among women with HDP, with linear trend lines for Period 1 (orange, solid) and Period 2 (blue, dashed). HDP = hypertensive disorders of pregnancy, PTB = preterm birth.

Despite the increasing prevalence of HDP, the proportion of pregnancies complicated by HDP that resulted in PTB declined from 13.9% in Period 1 to 13.5% in Period 2 (Figure 3B). Decomposition analysis estimated that this divergence corresponded to 89 fewer PTB with HDP cases than expected during Period 2 if Period 1 trends had persisted (Figure 3A, Table S2, Box S2).

Year‐by‐year decomposition demonstrated that PTB with HDP peaked in 2017 (749 cases, HDP prevalence 6.73%; PTB rate among women with HDP 14.51%). By 2021, although HDP prevalence had risen further, the PTB rate among affected pregnancies had declined to 12.70%. Although the cumulative burden of PTB with HDP remained elevated relative to the 2012 baseline due to increasing disease prevalence, observed case numbers were consistently lower than expected based on Period 1 trends (Table S6).

## 4 Discussion

In this population‐based time‐series study of 779,325 singleton births in Victoria between 2012 and 2022, we observed a divergence between increasing prevalence of HDP and declining PTB among affected pregnancies. The principal finding was a clear change in the temporal trend of PTB associated with HDP, with rates rising before mid‐2018 and declining thereafter. This trend reversal was driven predominantly by changes in iatrogenic PTB with HDP, whereas spontaneous PTB with HDP showed little evidence of temporal change across the study periods.

Decomposition analyses indicated that, despite rising HDP prevalence, the proportion of pregnancies complicated by HDP that resulted in PTB declined over time, corresponding to an estimated 89 (0.27 births per 1000 births) fewer cases than expected had the pre‐2018 patterns persisted. Taken together, these findings are consistent with a shift towards later gestational age at birth among pregnancies affected by HDP.

Several mechanisms may explain the observed shift towards later gestational age at birth among women with HDP. One possibility is a later gestational onset of HDP, potentially reflecting improved risk stratification through increasing use of effective first‐trimester screening and wider uptake of preventive strategies such as low‐dose aspirin, which is thought to delay disease onset rather than prevent HDP altogether [25]. Alternatively, HDP may be diagnosed at similar preterm gestational ages but with milder clinical severity, allowing affected pregnancies to be safely monitored and prolonged to term gestation. A third explanation is that the underlying disease profile has remained largely unchanged, but evolving clinical practice has resulted in more judicious use of preterm delivery, with greater emphasis on evidence‐based care and expectant management when maternal and foetal status permit [8]. Lastly, the introduction of angiogenic biomarker testing (sFlt‐1:PlGF ratio) in Victorian maternity services during Period 2 may have enhanced clinicians' ability to identify women with HDP at lower immediate risk, supporting safer expectant management and contributing to the observed trend of a later delivery timing [26].

These explanations are not mutually exclusive, and the observed trend reversal likely reflects a combination of improved preventive strategies, enhanced antenatal surveillance allowing safer expectant management and more judicious clinical decision‐making around the timing of delivery. We observed a sustained increase in PTB with HDP over several years that was followed by a relatively abrupt decline coinciding with the official launch of the national PTB prevention program in July 2018. This program placed renewed emphasis on the long‐term developmental consequences of PTB, which had previously been under‐recognised and promoted greater caution around medically indicated early delivery when pregnancy prolongation was clinically appropriate [27]. In contrast, biological mechanisms such as shifts in disease severity are less likely to account for the rapid change in temporal trends observed. Importantly, the absence of any increase in severe adverse pregnancy outcomes, including maternal ICU admission or stillbirth, suggests that later delivery among women with HDP was achieved without compromising maternal or foetal safety.

Our findings complement and extend the recent national evaluation of the Australian PTB prevention program [11]. While the national analysis demonstrated reductions in overall and late PTBs, it did not assess condition‐specific drivers or distinguish changes in disease prevalence from changes in obstetric management. By focusing on HDP, the leading indication for medically indicated PTB, and applying decomposition methods, we provide insight into the population‐level pathways through which national improvements may have been achieved.

Our results also align with global trends in HDP. A study from the Global Burden of Disease study showed that although the prevalence of HDP is increasing worldwide, the disability‐adjusted life years associated with HDP are declining in countries with high sociodemographic indices [28]. This suggests that our improvements in HDP‐associated PTB are part of the global improvements in practice implemented in high‐income countries.

Our findings also help clarify the widely held view that the COVID‐19 pandemic was associated with population‐level reductions in PTB. Early reports from multiple jurisdictions, including our own work from metropolitan Melbourne, identified reductions in PTB during periods of lockdown [29, 30, 31]. However, many such studies relied on short baseline periods, single centre cohorts, simple before–after comparisons or did not formally account for underlying secular trends and seasonality. In contrast, our present population‐based study incorporates an 8‐year pre‐pandemic baseline, formal time‐series modelling accounting for autocorrelation and seasonality, and objective identification of inflection points. Using this approach, we demonstrate that reductions in PTB observed during the pandemic period represented a continuation of trends that began before 2020, and the pandemic‐specific effect may have accelerated or contributed to the existing trend. Although our analysis did not identify a distinct inflection point coinciding with pandemic onset, we cannot exclude the possibility that pandemic‐related factors may have accelerated or reinforced the existing downward trajectory in PTB among women with HDP.

Key strengths of our study include the whole‐of‐population design, capturing all singleton public and private hospital births in Victoria over a decade, and the use of SARIMA modelling to account for seasonality and autocorrelation. Our decomposition analyses allowed separation of changes in disease prevalence from changes in clinical management, providing the first population‐based Australian data to examine the changing disease burden of HDP. Limitations include reliance on administrative data that lacks individual information on body mass index, aspirin use, prior obstetric history, gestational age at HDP onset and HDP severity beyond ICU admission and stillbirth, as well as the inability to separate the effects of different possible practice changes that occurred during the study period. Changes in diagnostic coding precluded separation of gestational hypertension from pre‐eclampsia during the study period. Non‐random shifts in ICD‐10‐AM coding for HDP subtypes (chronic hypertension, gestational hypertension, pre‐eclampsia/eclampsia and unspecified maternal hypertension) over the study period meant that any subtype‐specific temporal trend in these data would conflate genuine epidemiological change with administrative reclassification. Subtype‐specific analyses, including evaluation of differential effects of preventive strategies such as low‐dose aspirin across HDP subtypes, will require linked clinician‐reported data such as the Victorian Perinatal Data Collection and are a priority for future work. Finally, our findings may not be generalisable to settings with different models of maternity care.

To quantify specific contributions to observed trends, future research should include individual‐level data on aspirin use, pre‐eclampsia screening uptake and gestational age at HDP onset. Research should also identify barriers to implementation in disadvantaged communities and public hospitals and evaluate whether later births of infants for mothers with HDP have improved maternal and neonatal outcomes.

## 5 Conclusion

In Victoria, HDP became more common over time, but PTB among women with HDP declined. This reduction in PTB among HDP‐affected pregnancies appears to have offset the rising disease burden, resulting in fewer PTBs than expected. These findings suggest that reductions in PTB are achievable even as major risk factors such as HDP become more prevalent.

## Author Contributions

**Melvin Marzan:** conceptualization, methodology, software, validation, data curation, formal analysis, visualization, and writing – original draft and funding acquisition; **Lisa Hui:** conceptualization, validation, data curation, visualization, writing – review and editing, funding acquisition and supervision; **Daniel Lorber Rolnik:** conceptualization, validation, data curation, formal analysis, writing – review and editing and funding acquisition; **Heng Jiang:** conceptualisation, validation, formal analysis, writing – review and editing, and funding acquisition; **Joanne M. Said:** validation, resources, formal analysis, and writing – review and editing.

## Funding

The authors have nothing to report.

## Disclosure

Not commissioned; externally peer reviewed.

## Conflicts of Interest

The authors declare no conflicts of interest.

## Supporting information


**Table S1:** STROBE checklist.
**Box S1**. Key Strategies of the Australian preterm birth prevention program.
**Figure S1:** Monthly trends in preterm birth with and without HDP, stratified by gestational age, Victoria 2012–2022.
**Table S2:** Year‐by‐year decomposition analysis of preterm birth with hypertensive disorders of pregnancy, Victoria, Australia (2012–2021).
**Table S3:** Period‐level decomposition analysis showing derivation of prevented cases.
**Table S4:** Subgroup SARIMA analysis.
**Table S5:** Seasonal autoregressive integrated moving average (SARIMA) analysis of preterm birth and hypertensive disorders of pregnancy trends by study period with *p*‐value.
**Figure S2:** Monthly trends in preterm birth with and without HDP, stratified by remoteness, Victoria 2012–2022.
**Figure S3:** Monthly trends in preterm birth with and without HDP, stratified by Hospital Type, Victoria 2012–2022.
**Figure S4:** Monthly trends in preterm birth with and without HDP, stratified by socioeconomic status, Victoria 2012–2022.
**Figure S5:** Diagnostic.
**Box S2**. Period‐level decomposition analysis showing derivation of prevented cases.
**Table S6:** Sensitivity analysis of seasonal autoregressive integrated moving average (SARIMA) analysis of preterm birth and hypertensive disorders of pregnancy trends by study period excluding COVID‐19 lockdown periods (April 2020‐onwards).

## Data Availability

The data that underlie the findings of our study are available upon reasonable request to researchers at approved research institutes, subject to approval from the VAED data custodian in the Victorian Department of Health.
