# Supplementary material for: Temporal Trends in Preterm Birth Associated With Hypertensive Disorders of Pregnancy in Victoria, Australia: A Population‐Based Interrupted Time‐Series Study
Source: Med J Aust. 2026 Aug 24;224(8):e70267. doi: 10.5694/mja2.70267 (PMC13502304; doi:10.5694/mja2.70267)
Supplement: Supplementary file 1 [file MJA2-224-0-s001.pdf]

Supporting Information

Supplementary material

This appendix was part of the submitted manuscript and has been peer reviewed.
It is posted as supplied by the authors.

Appendix to: Marzan M, Jiang H, Rolnik DL, et al. Temporal trends in preterm birth associated with hypertensive disorders of pregnancy in Victoria, Australia: a population-based interrupted time-series study. *Med J Aust* 2026; doi: 10.5694/mja2.70267.

Table S1: STROBE Checklist

	Item No	Recommendation	Page number
**Title and abstract**	1	(*a*) Indicate the study's design with a commonly used term in the title or the abstract	1
		(*b*) Provide in the abstract an informative and balanced summary of what was done and what was found	1
**Introduction**			
Background/rationale	2	Explain the scientific background and rationale for the investigation being reported	3 – 4
Objectives	3	State specific objectives, including any prespecified hypotheses	4
**Methods**			
Study design	4	Present key elements of study design early in the paper	4
Setting	5	Describe the setting, locations, and relevant dates, including periods of recruitment, exposure, follow-up, and data collection	4
Participants	6	(*a*) Give the eligibility criteria, and the sources and methods of selection of participants. Describe methods of follow-up	4-5
		(*b*) For matched studies, give matching criteria and number of exposed and unexposed	n/a
Variables	7	Clearly define all outcomes, exposures, predictors, potential confounders, and effect modifiers. Give diagnostic criteria, if applicable	4-6
Data sources/ measurement	8*	For each variable of interest, give sources of data and details of methods of assessment (measurement). Describe comparability of assessment methods if there is more than one group	*4-5*
Bias	9	Describe any efforts to address potential sources of bias	7
Study size	10	Explain how the study size was arrived at	n/a
Quantitative variables	11	Explain how quantitative variables were handled in the analyses. If applicable, describe which groupings were chosen and why	6-7
Statistical methods	12	(*a*) Describe all statistical methods, including those used to control for confounding	6-7
		(*b*) Describe any methods used to examine subgroups and interactions	6-7
		(*c*) Explain how missing data were addressed	n/a
		(*d*) If applicable, explain how loss to follow-up was addressed	n/a
		(*e*) Describe any sensitivity analyses	n/a
**Results**			
Participants	13*	(a) Report numbers of individuals at each stage of study—eg numbers potentially eligible, examined for eligibility, confirmed eligible, included in the study, completing follow-up, and analysed	
		(b) Give reasons for non-participation at each stage	
		(c) Consider use of a flow diagram	
Descriptive data	14*	(a) Give characteristics of study participants (eg demographic, clinical, social) and information on exposures and potential confounders	8
		(b) Indicate number of participants with missing data for each variable of interest	n/a
		(c) Summarise follow-up time (eg, average and total amount)	n/a
Outcome data	15*	Report numbers of outcome events or summary measures over time	8-9
Main results	16	(*a*) Give unadjusted estimates and, if applicable, confounder-adjusted estimates and their precision (eg, 95% confidence interval). Make clear which confounders were adjusted for and why they were included	8-9<br>Tables 1-3
		(*b*) Report category boundaries when continuous variables were categorized	8-9<br>Tables 1-3

		(*c*) If relevant, consider translating estimates of relative risk into absolute risk for a meaningful time period	n/a
Other analyses	17	Report other analyses done—eg analyses of subgroups and interactions, and sensitivity analyses	
**Discussion**			
Key results	18	Summarise key results with reference to study objectives	9
Limitations	19	Discuss limitations of the study, taking into account sources of potential bias or imprecision. Discuss both direction and magnitude of any potential bias	12-13
Interpretation	20	Give a cautious overall interpretation of results considering objectives, limitations, multiplicity of analyses, results from similar studies, and other relevant evidence	9-13
Generalisability	21	Discuss the generalisability (external validity) of the study results	13
**Other information**			
Funding	22	Give the source of funding and the role of the funders for the present study and, if applicable, for the original study on which the present article is based	n/a

Box S1: Key Strategies of the Australian preterm birth prevention program [1]

- No pregnancy to be ended until at least 39 weeks unless there is obstetric or medical justification.
- Measurement of the length of the cervix at all mid-pregnancy scans.
- Use of natural vaginal progesterone (200mg each evening) if the length of cervix is less than 25mm.
- If the length of the cervix continues to shorten despite progesterone treatment, consider surgical cerclage.
- Use of vaginal progesterone if there is a prior history of spontaneous preterm birth.
- Women who smoke should be identified and offered Quitline support.
- To access continuity of care from a known midwife during pregnancy where possible.

Supplementary Methods – Joinpoint regression.

Joinpoint regression was applied to the same monthly outcome series (cases per 1,000 singleton births) as the main SARIMA framework, as a data-driven sensitivity analysis of the timing of trajectory change. Each series was first seasonally adjusted using seasonal-trend decomposition based on loess (STL) [2], retaining the trend plus remainder as the analytical series. Piecewise linear regression was then fitted using the segmented R package [3], which estimates joinpoint locations and segment-specific slopes by iteratively solving a constrained linear model. Candidate models with zero, one, two, and three joinpoints were fitted with initial joinpoint locations spread evenly across the series, and the best-fitting model was selected by minimum Bayesian Information Criterion. For each segment we report the slope per 1,000 singleton births per month, 95% confidence interval, and p-value derived from the segmented fit.

Figure S1: Monthly trends in preterm birth with and without HDP, stratified by gestational age, Victoria 2012-2022

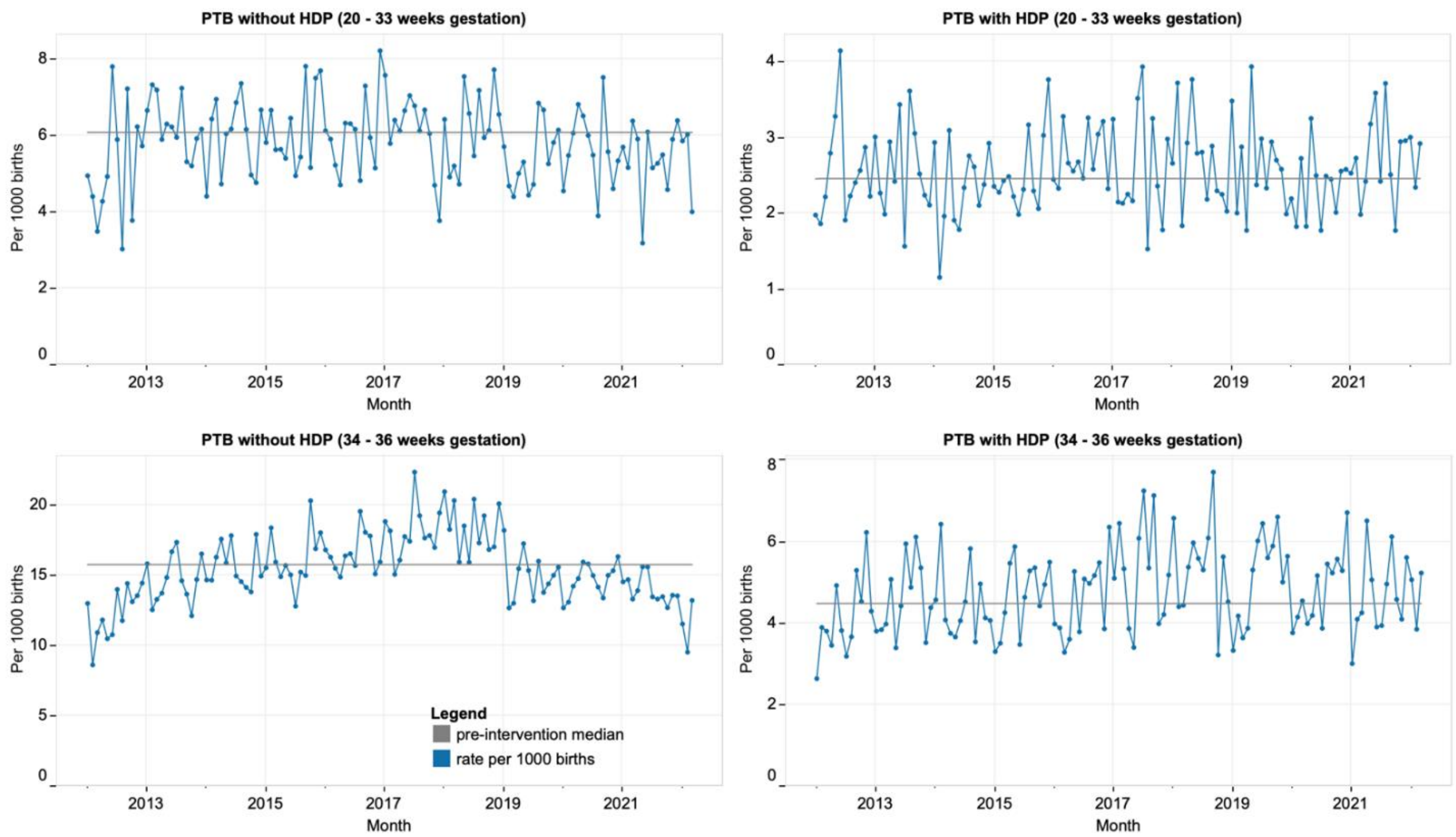

Abbreviations: HDP = hypertensive disorders of pregnancy; PTB = preterm birth.

Table S2. Year-by-year decomposition analysis of preterm birth with hypertensive disorders of pregnancy, Victoria, Australia (2012-2021)

Year	Period	Total births	HDP cases	HDP prevalence	PTB with HDP (observed)	PTB|HDP rate (%)	PTB with HDP (expected)*	Cumulative offset†
2012	Period 1	74,474	4,556	6.12%	631	13.85	631	0
2013	Period 1	74,731	4,828	6.46%	643	13.32	633	-10
2014	Period 1	75,951	4,629	6.10%	630	13.61	643	4
2015	Period 1	76,096	4,753	6.25%	684	14.39	645	-36
2016	Period 1	77,803	5,069	6.52%	698	13.77	660	-74
2017	Period 1	76,689	5,161	6.73%	749	14.51	650	-174
2018	Period 2	75,641	5,132	6.78%	724	14.11	641	-257
2019	Period 2	76,145	5,199	6.83%	745	14.33	645	-357
2020	Period 2	74,367	5,439	7.31%	689	12.67	630	-415
2021	Period 2	78,629	5,731	7.29%	728	12.70	666	-477

Abbreviations: HDP = hypertensive disorders of pregnancy; PTB = preterm birth; PTB|HDP = rate of preterm birth among women with HDP

NOTES:

- *Expected PTB with HDP calculated by applying 2012 baseline rates (HDP prevalence 6.12%, PTB|HDP rate 13.85%) to each year's total births.
- †Cumulative offset = running sum of annual differences between expected and observed PTB with HDP cases from 2012 baseline.
- Negative values indicate more cases than expected; positive values indicate fewer cases than expected.
- The cumulative burden worsened from -174 cases in 2017 (Period 1) to -477 cases by 2021, reflecting ongoing increases in HDP prevalence (6.12% in 2012 to 7.29% in 2021).
- However, the rate of PTB among women with HDP declined from 14.51% in 2017 to 12.70% in 2021, indicating improvements in clinical management despite rising disease prevalence.
- Note: This year-by-year decomposition uses 2012 as a fixed baseline and differs from the period-level decomposition reported in the main text (89 fewer cases, Table S3), which compares the Period 1 average (Jan 2012–Jun 2018, 13.89% rate) to the Period 2 (Jul 2018–Mar 2022, 13.51% rate).

Table S3. Period-level decomposition analysis showing derivation of prevented cases

Period/Component	Total births	HDP cases	HDP prevalence	PTB with HDP (observed)	PTB|HDP rate (%)	PTB with HDP (expected)	Prevented cases
**Period 1 (Jan 2012 - Jun 2018)**	449,373	28,564	6.36%	3,968	13.89%	—	—
**Period 2 (Jul 2018 – Mar 2022)**	329,952	23,157	7.02%	3,128	13.51%	3,217*	89†
**Decomposition Analysis**	—	—	+0.66 pp	—	-0.38 pp	—	—

Abbreviations: HDP = hypertensive disorders of pregnancy; PTB = preterm birth; PTB|HDP = rate of preterm birth among women with HDP; pp = percentage points.

* Expected PTB with HDP = Period 2 HDP cases × Period 1 PTB|HDP rate (23,157 × 13.89% = 3,217 cases).

† Prevented cases = expected minus observed PTB with HDP (3,217 − 3,128 = 89 fewer cases than expected).

Table S4. Subgroup SARIMA Analysis

Indicator	Pre-reference Period Slope Jan 2012 – Jun 2018	Change in Slope	Period 2 Slope (95% CI) Jul 2018 – Mar 2022[a]
**Gestational age (weeks)**			
iPTB with HDP (20 – 33 weeks GA)	0.074 (-0.010, 0.159)	-0.093 (-0.291, 0.105)	-0.019 (-0.160 to 0.122)
iPTB without HDP (20 – 33 weeks GA)	0.037 (0.006, 0.069)*	-0.028 (-0.113, 0.057)	0.009 (-0.053 to 0.072)
iPTB with HDP (34 – 36 weeks GA)	0.175 (-0.047, 0.397)	-0.308 (-0.770, 0.155)	-0.132 (-0.450 to 0.185)
iPTB without HDP (34 – 36 weeks GA)	0.062 (-0.000, 0.125)	-0.077 (-0.224, 0.070)	-0.014 (-0.120 to 0.091)
**Remoteness**			
iPTB with HDP (Regional)	0.022 (0.009, 0.034)***	-0.024 (-0.067, 0.019)	-0.003 (-0.038 to 0.033)
iPTB without HDP (Regional)	0.063 (0.027, 0.099)***	-0.093 (-0.200, 0.015)	-0.029 (-0.112 to 0.053)
iPTB with HDP (Metropolitan)	0.074 (-0.011, 0.159)	-0.084 (-0.263, 0.095)	-0.010 (-0.135 to 0.115)
iPTB without HDP (Metropolitan)	0.171 (-0.082, 0.423)	-0.278 (-0.743, 0.188)	-0.107 (-0.432 to 0.218)
**Type of hospital**			
iPTB with HDP (Private)	0.024 (0.010, 0.039)***	-0.024 (-0.071, 0.022)	0.000 (-0.037 to 0.038)
iPTB without HDP (Private)	0.051 (-0.021, 0.124)	-0.067 (-0.209, 0.076)	-0.015 (-0.117 to 0.087)
iPTB with HDP (Public)	0.066 (-0.023, 0.155)	-0.067 (-0.243, 0.109)	-0.001 (-0.123 to 0.121)
iPTB without HDP (Public)	0.180 (-0.089, 0.449)	-0.305 (-0.823, 0.214)	-0.124 (-0.474 to 0.225)
**Socioeconomic status**			
iPTB with HDP (more disadvantaged)	0.061 (0.014, 0.109)*	-0.067 (-0.203, 0.069)	-0.005 (-0.108 to 0.097)
iPTB without HDP (more disadvantaged)	0.183 (0.058, 0.309)**	-0.329 (-0.694, 0.035)	-0.146 (-0.419 to 0.127)
iPTB with HDP (more advantaged)	0.046 (0.008, 0.083)*	-0.054 (-0.156, 0.047)	-0.009 (-0.084 to 0.067)
iPTB without HDP (more advantaged)	0.101 (-0.060 to 0.262)	-0.133 (-0.469 to 0.203)	-0.032 (-0.260 to 0.195)

Abbreviations: CI = confidence interval; GA = gestational age; HDP = hypertensive disorders of pregnancy; iPTB = iatrogenic preterm birth; PTB = preterm birth; SARIMA = seasonal autoregressive integrated moving average.

Coefficients represent absolute monthly change in rate per 1 000 births | *<0.05, **<0.01, ***<0.001

[a] Period 1 slope (β_t) represents the baseline trend. The Change in Slope (β_{ramp1}) represents the shift in trajectory following the intervention. The Period 2 Slope is the absolute trend for the second period, calculated as the linear combination of the baseline slope and the change: Period 2 Slope = $\beta_t + \beta_{ramp1}$.

Table S5. Seasonal autoregressive integrated moving average (SARIMA) analysis of preterm birth and hypertensive disorders of pregnancy trends by study period with p-value

Primary and secondary outcomes	Period 1 Slope (95% CI)<br>Jan 2012 – Jun 2018	Change in Slope (95% CI)	Period 2 Slope (95% CI)<br>Jul 2018 – Mar 2022[a]
**PTB with HDP**	0.751 (0.503 to 0.999)	-1.122 (-1.884 to -0.360)**	-0.371 (0.946 to 0.205)
p-value	0.000	0.00391	0.20734
Iatrogenic PTB with HDP	0.100 (0.008 to 0.191)*	-0.086 (-0.310 to 0.138)	0.014 (-0.148 to 0.175)
p-value	0.0328	0.45204	0.86781
Spontaneous PTB with HDP	0.018 (-0.016 to 0.052)	-0.014 (-0.101 to 0.074)	0.004 (-0.057 to 0.065)
p-value	0.3038	0.76037	0.89136
**Total PTB**	0.518 (-0.549 to 1.585)	-0.364 (-2.514 to 1.785)	0.154 (-1.294 to 1.602)
p-value	0.3410	0.73969	0.83512
Total iatrogenic PTB	0.341 (-0.031 to 0.713)	-0.420 (-1.203 to 0.363)	-0.079 (-0.622 to 0.465)
p-value	0.0723	0.29352	0.77710
Total spontaneous PTB	0.048 (-0.409 to 0.505)	0.102 (-0.605 to 0.809)	0.150 (-0.401 to 0.700)
p-value	0.8376	0.77780	0.59431
**Total HDP**	0.405 (-0.440 to 1.250)	-0.103 (-1.408 to 1.203)	0.302 (-0.685 to 1.290)
p-value	0.3473	0.87758	0.54835
Term pregnancies with HDP	0.027 (0.005 to 0.048)*	-0.011 (-0.050 to 0.029)	0.016 (-0.013 to 0.044)
p-value	0.0175	0.58911	0.27754
Term pregnancies without HDP	0.120 (0.106 to 0.134)***	-0.177 (-0.244 to -0.110)***	-0.057 (-0.124 to 0.010)
p-value	0.0000	0.00000	0.09480
**PTB without HDP**	0.097 (-0.041 to 0.236)	-0.073 (-0.364 to 0.218)	0.025 (-0.177 to 0.227)
p-value	0.1673	0.62426	0.81096
Iatrogenic PTB without HDP	0.220 (-0.085 to 0.526)	-0.365 (-0.934 to 0.204)	-0.145 (-0.541 to 0.251)
p-value	0.1579	0.20880	0.47413
Spontaneous PTB without HDP	0.058 (-0.381 to 0.497)	0.076 (-0.608 to 0.759)	0.134 (-0.393 to 0.660)
p-value	0.3038	0.76037	0.89136

Abbreviations: CI = confidence interval; HDP = hypertensive disorders of pregnancy; PTB = preterm birth; SARIMA = seasonal autoregressive integrated moving average.

Coefficients represent absolute monthly change in rate per 1 000 births | *<0.05, **<0.01, ***<0.001

[a] Period 1 slope (β_t) represents the baseline trend. The Change in Slope (β_{ramp1}) represents the shift in trajectory following the intervention. The Period 2 Slope is the absolute trend for the second period, calculated as the linear combination of the baseline slope and the change: Period 2 Slope = $\beta_t + \beta_{ramp1}$.

Figure S2: Monthly trends in preterm birth with and without HDP, stratified by remoteness, Victoria 2012-2022

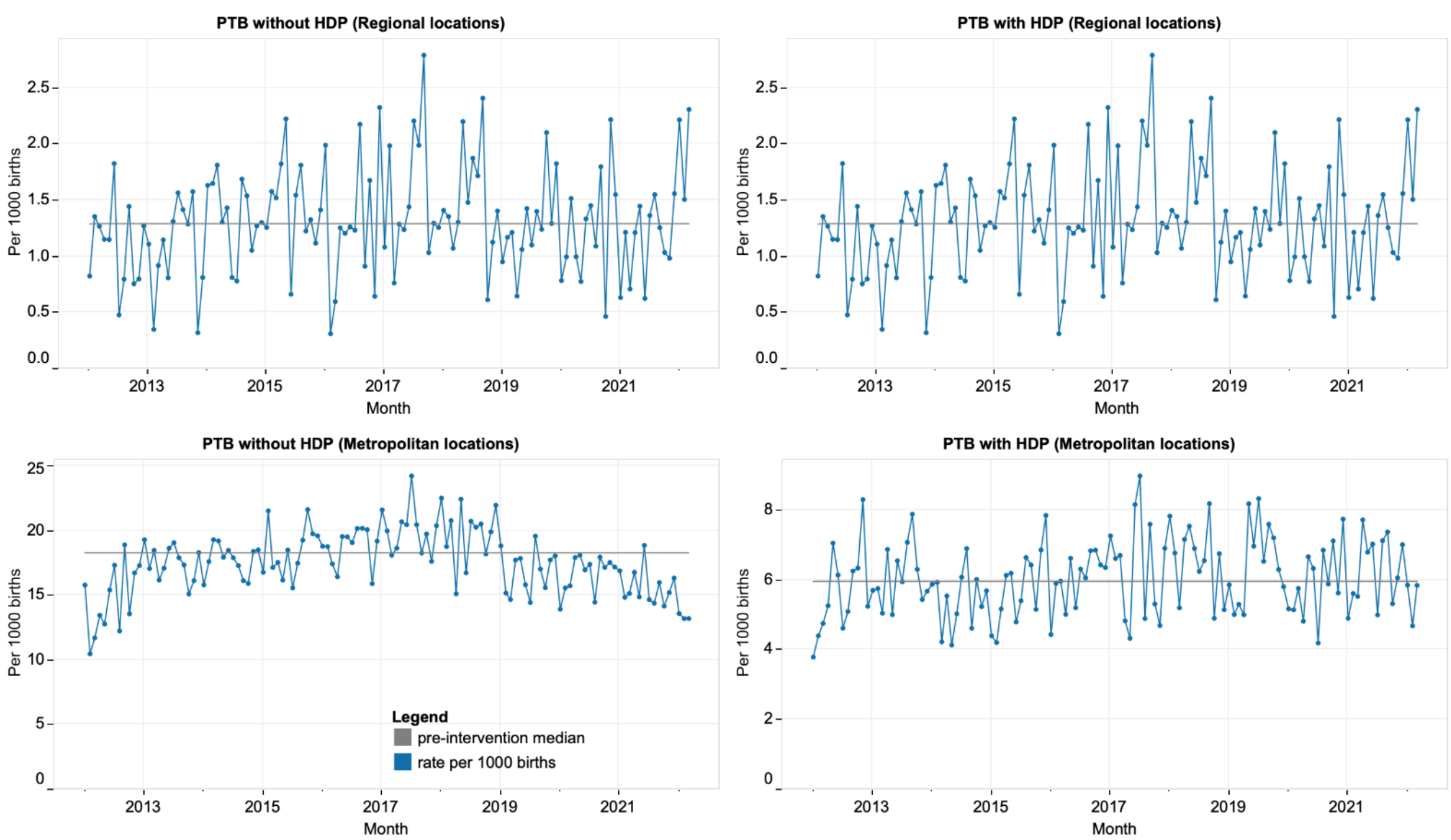

Abbreviations: HDP = hypertensive disorders of pregnancy; PTB = preterm birth.

Figure S3: Monthly trends in preterm birth with and without HDP, stratified by Hospital Type, Victoria 2012-2022

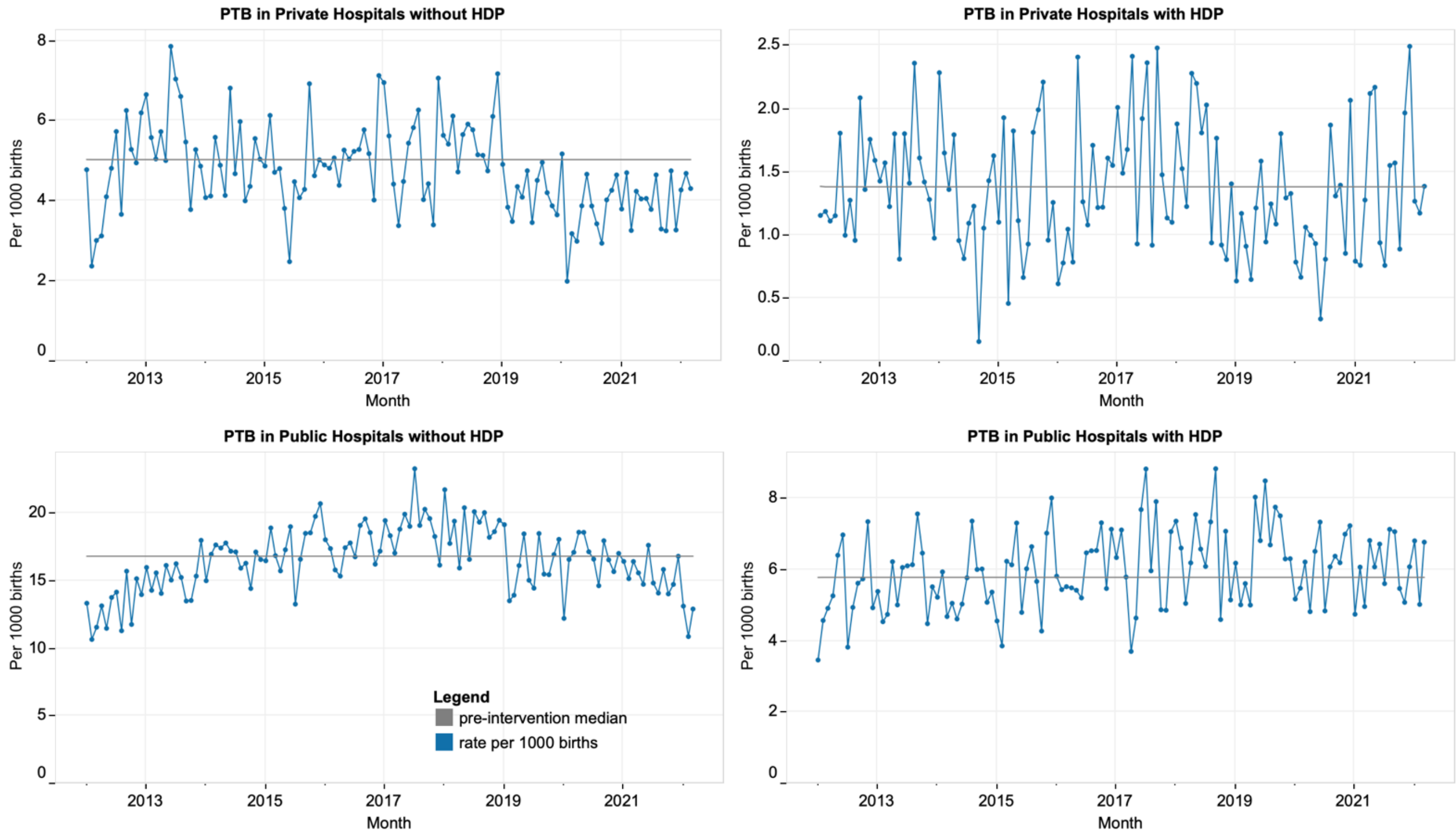

Abbreviations: HDP = hypertensive disorders of pregnancy; PTB = preterm birth.

Figure S4: Monthly trends in preterm birth with and without HDP, stratified by socioeconomic status, Victoria 2012-2022

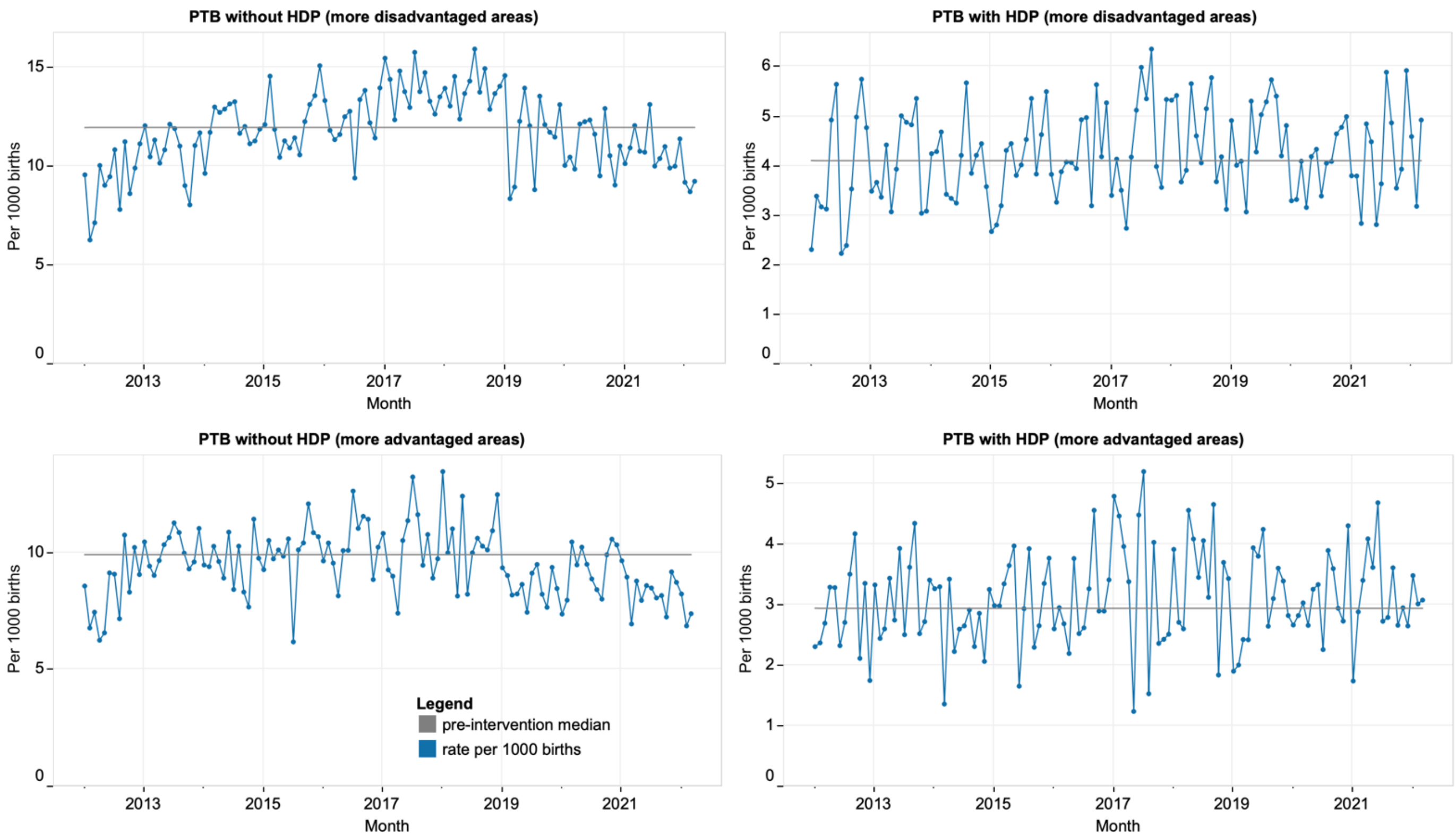

Abbreviations: HDP = hypertensive disorders of pregnancy; PTB = preterm birth; SEIFA = Socio-Economic Indexes for Areas.

Figure S5: Diagnostic Plots for SARIMA

Abbreviations: ACF = autocorrelation function; GA = gestational age; HDP = hypertensive disorders of pregnancy; PACF = partial autocorrelation function; PTB = preterm birth; SARIMA = seasonal autoregressive integrated moving average; SEIFA = Socio-Economic Indexes for Areas.

A. Overall HDP

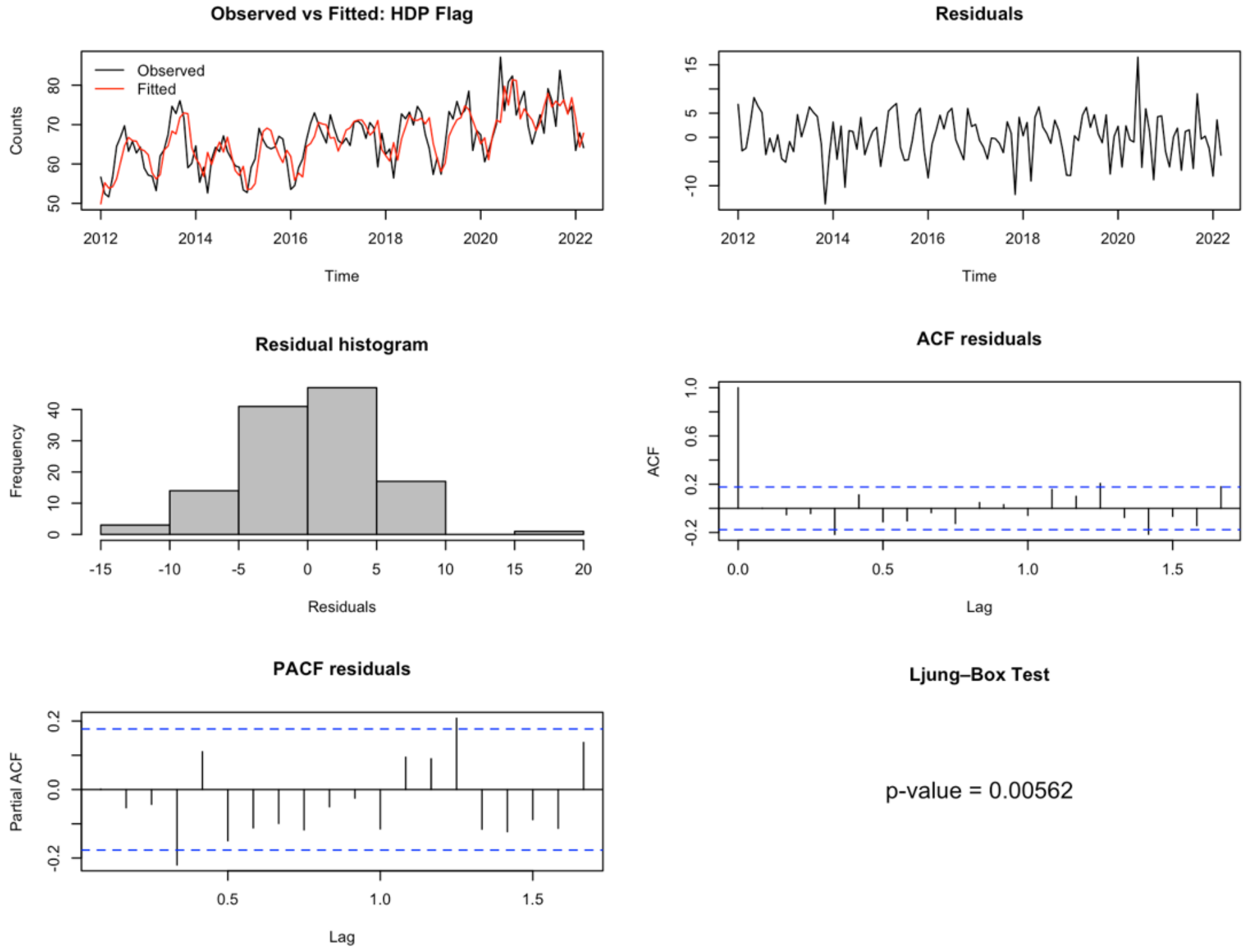

B. Overall PTB with HDP

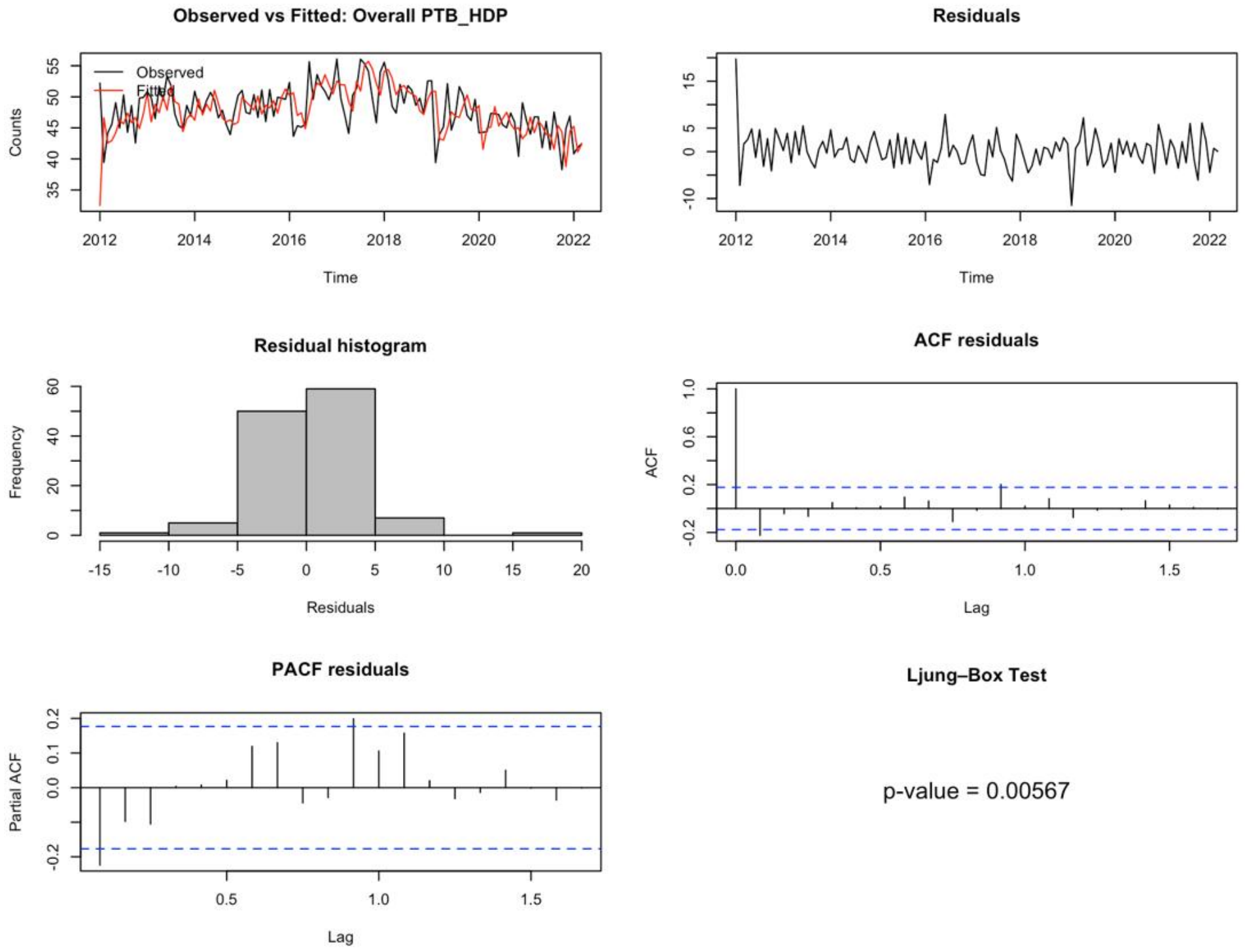

C. Iatrogenic PTB

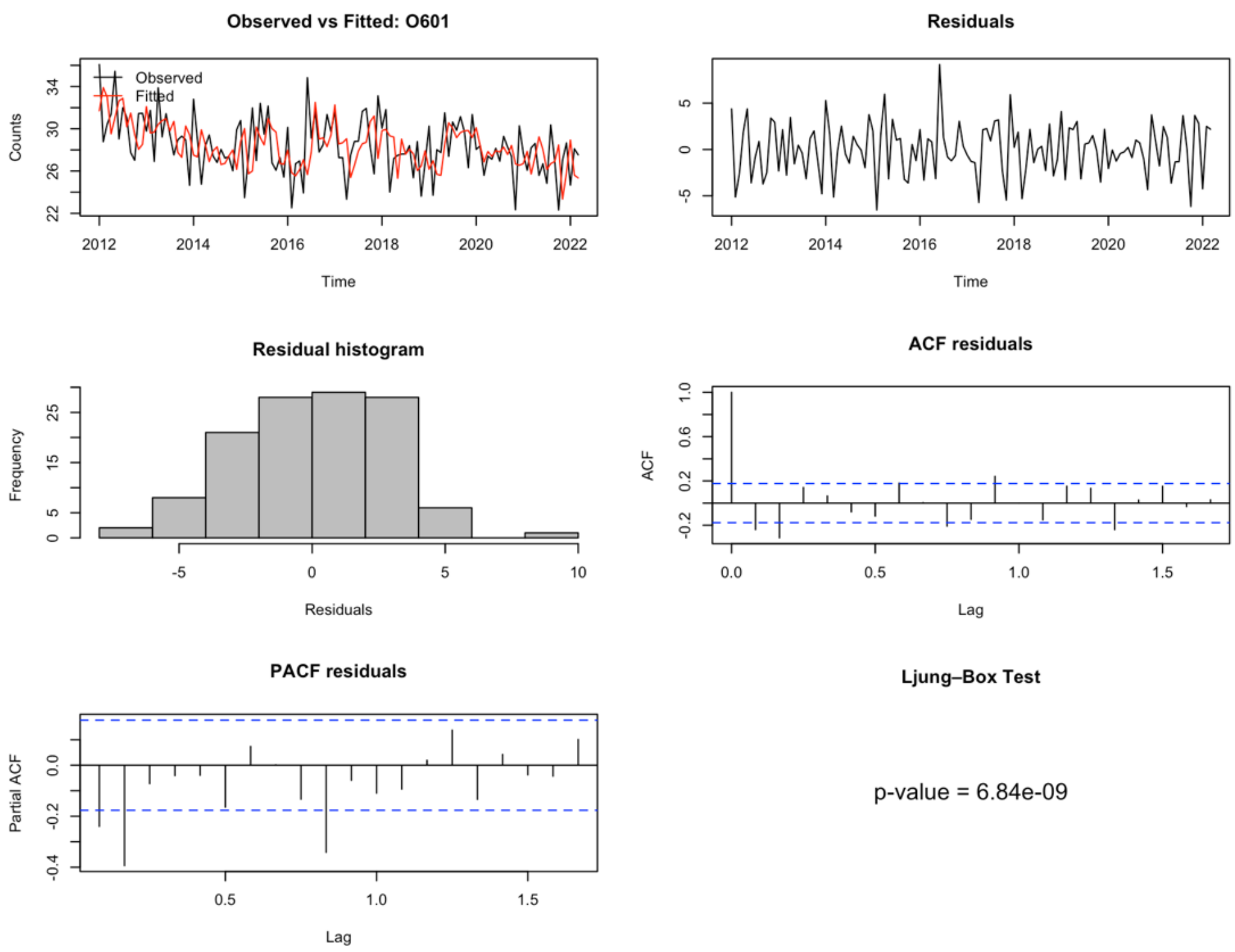

D. Spontaneous PTB

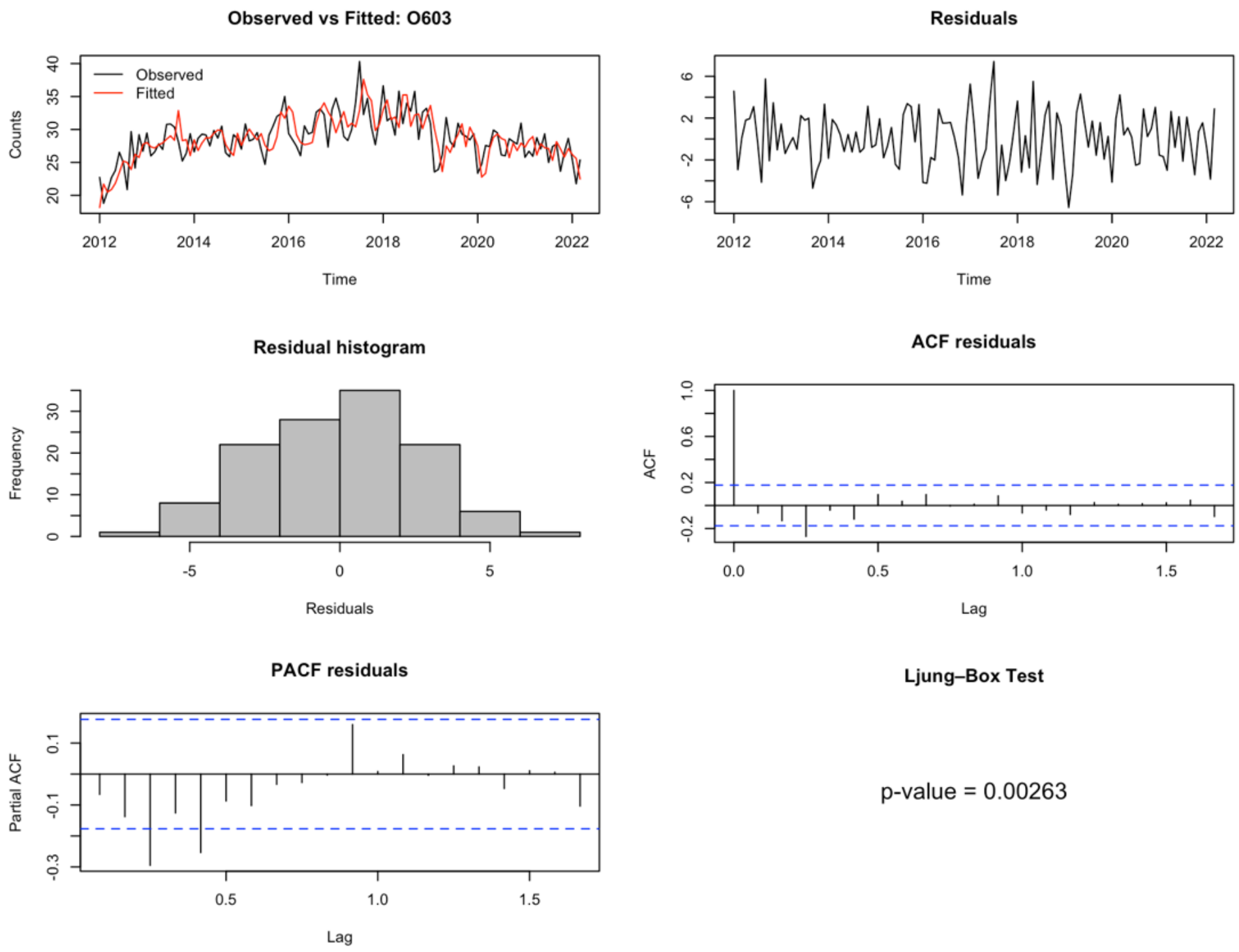

E. Overall PTB with HDP

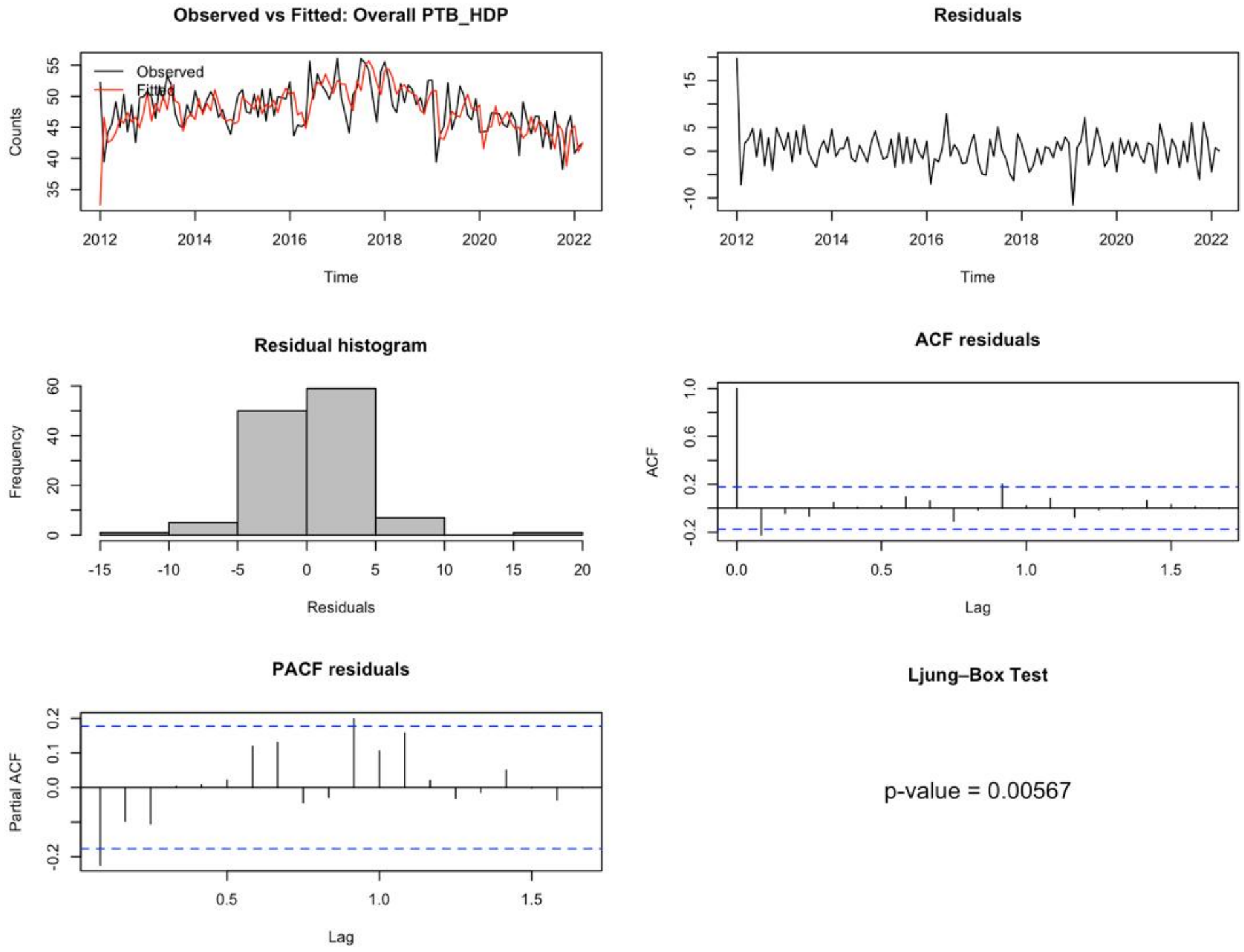

F. Overall PTB without HDP

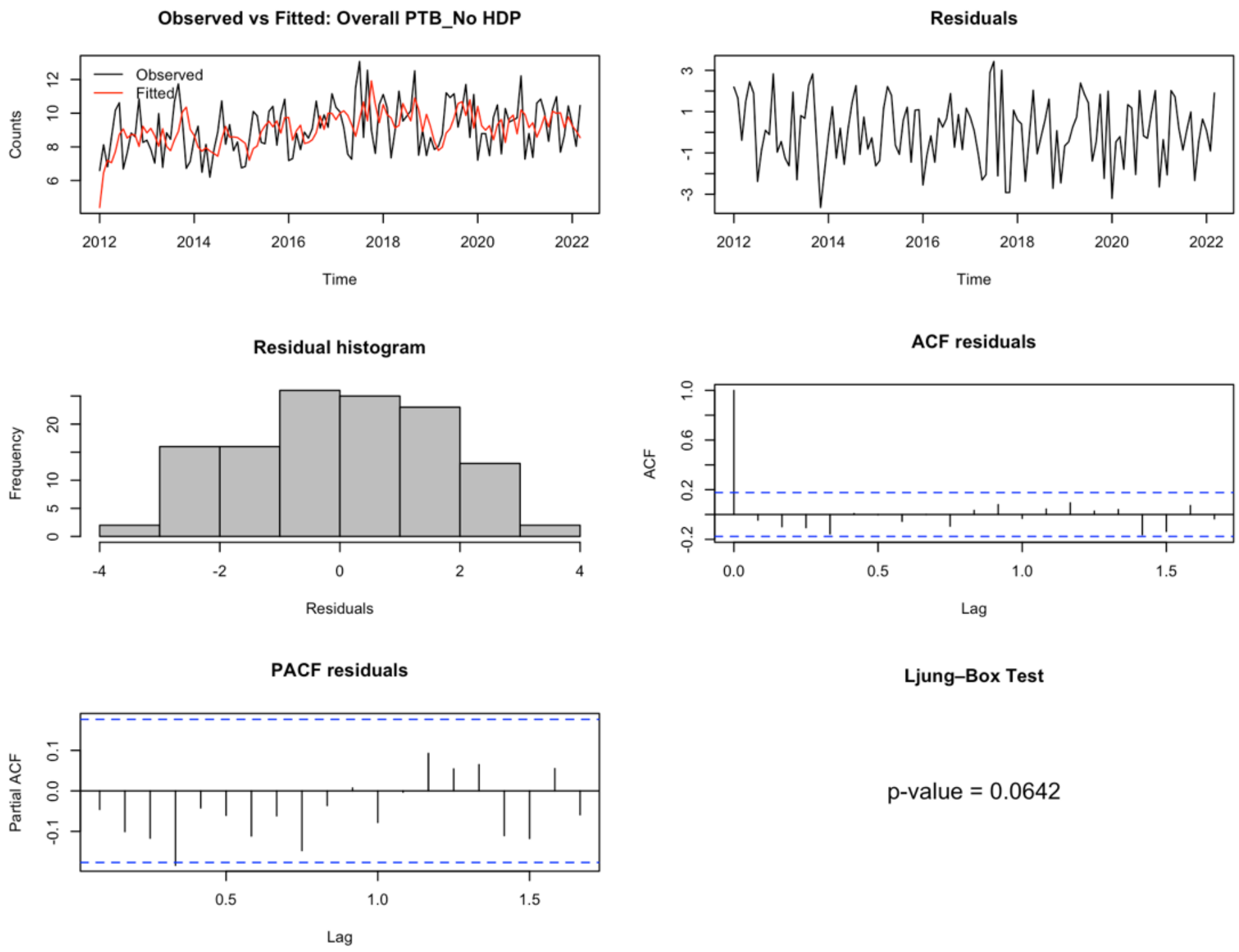

G. Iatrogenic PTB with HDP

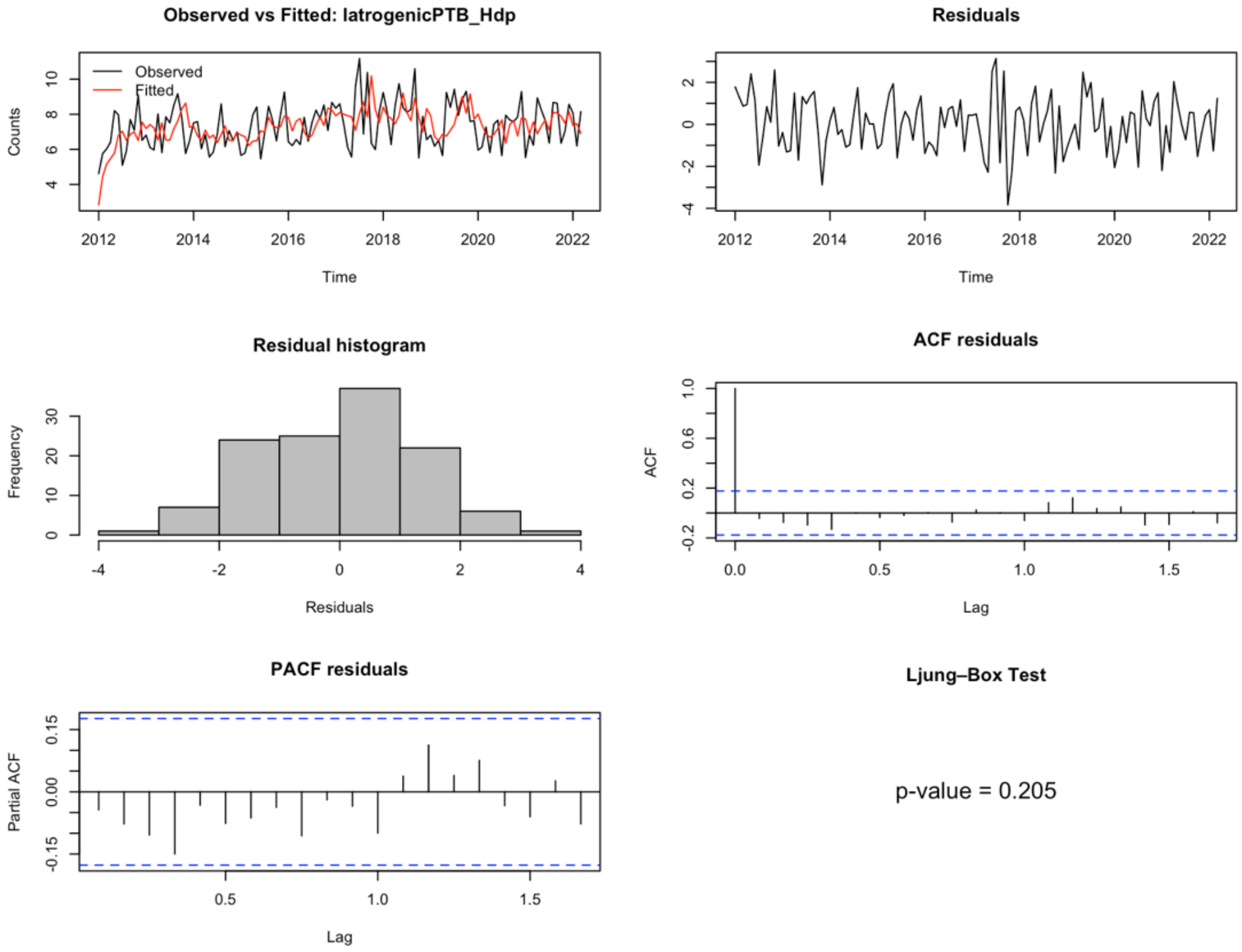

H. Iatrogenic PTB without HDP

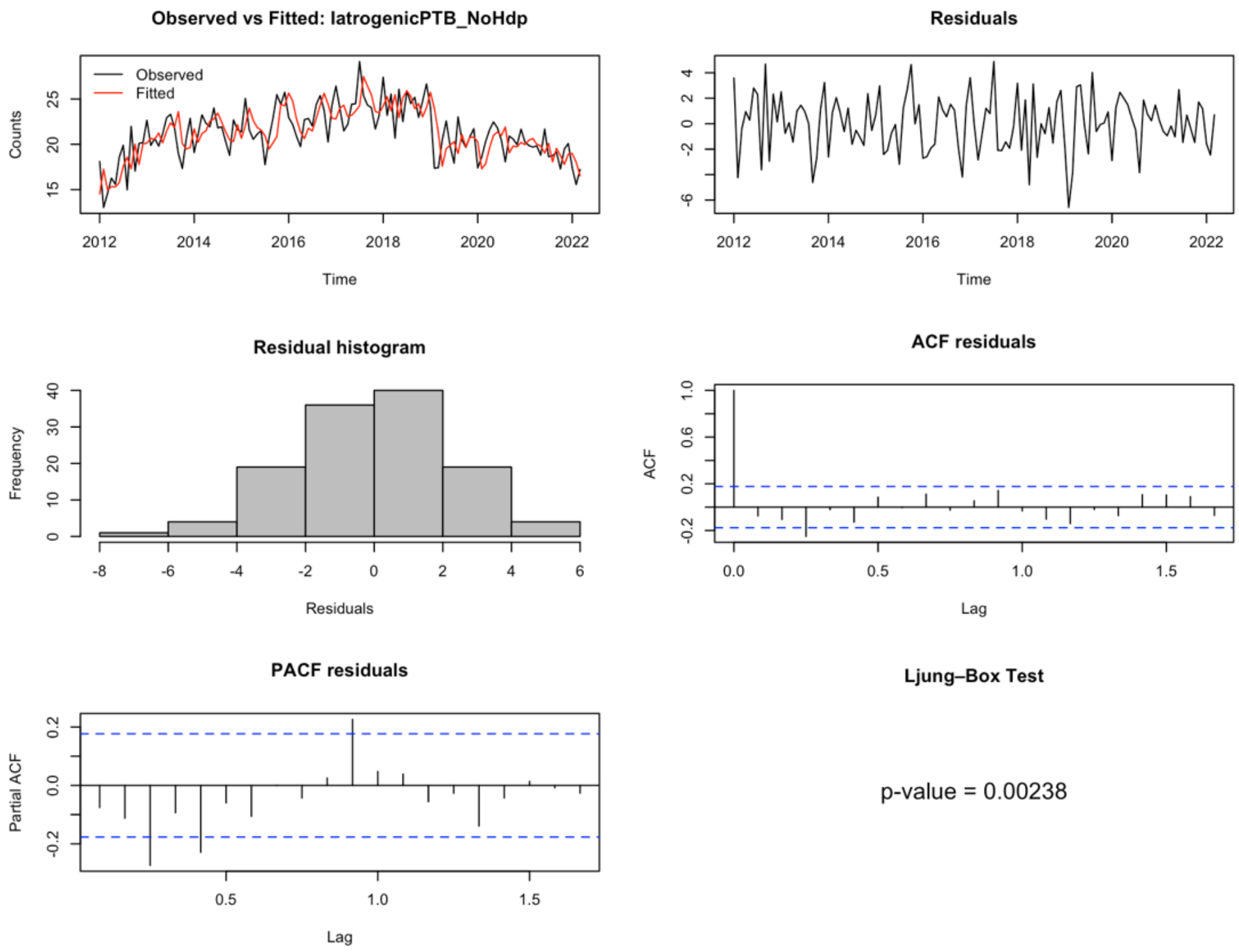

I. 20-30 Weeks GA with HDP

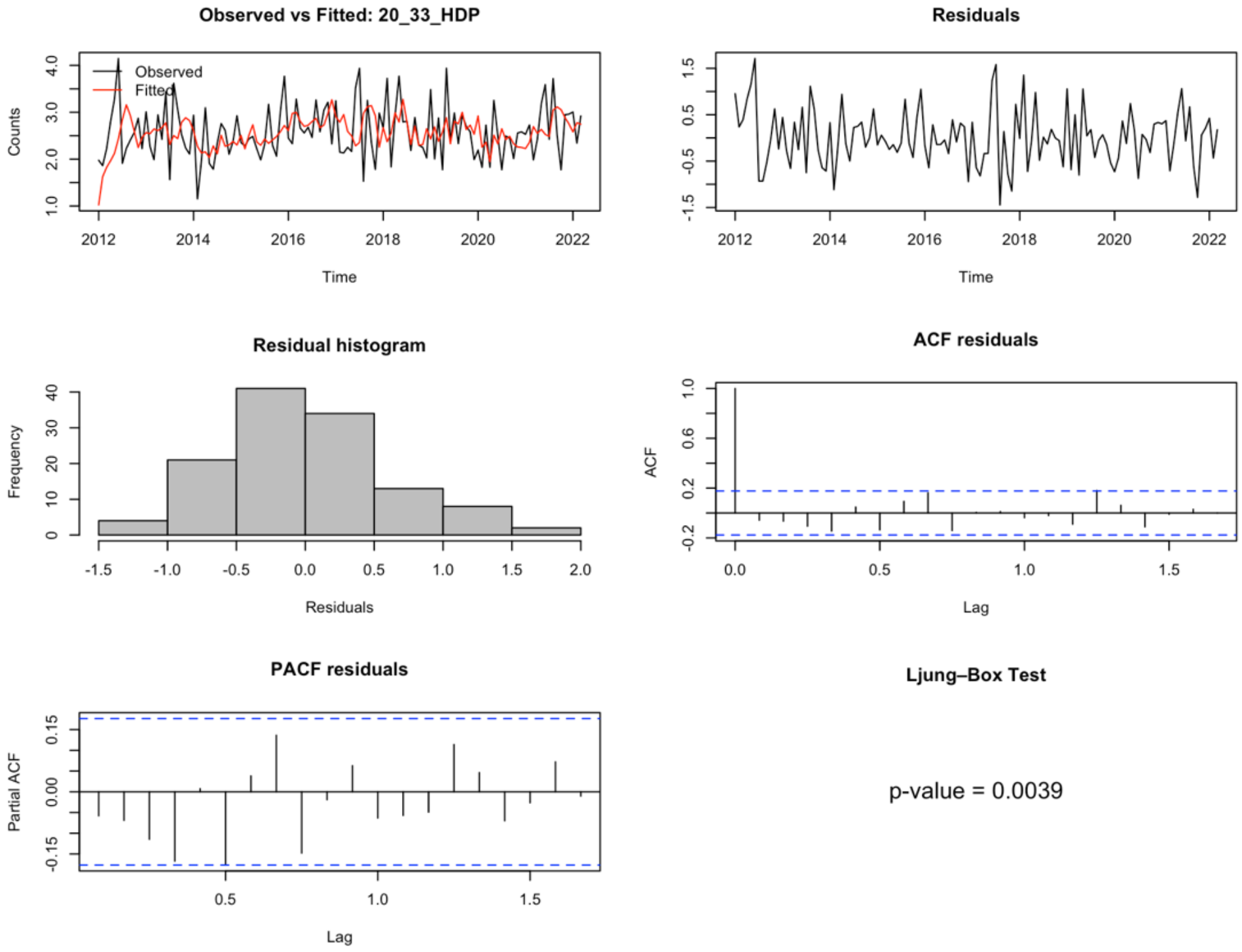

J. 20-30 Weeks GA without HDP

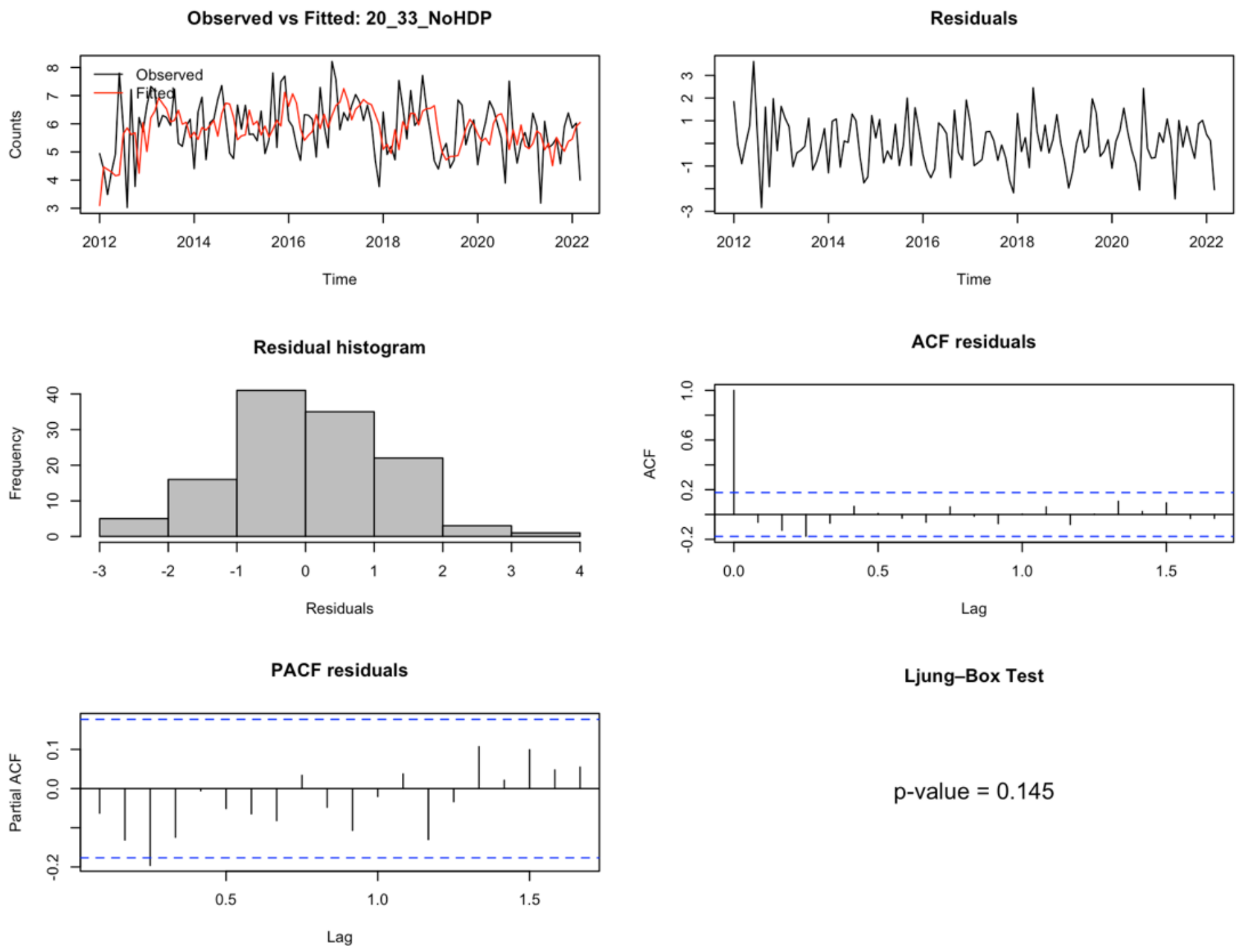

K. 34-36 Weeks GA with HDP

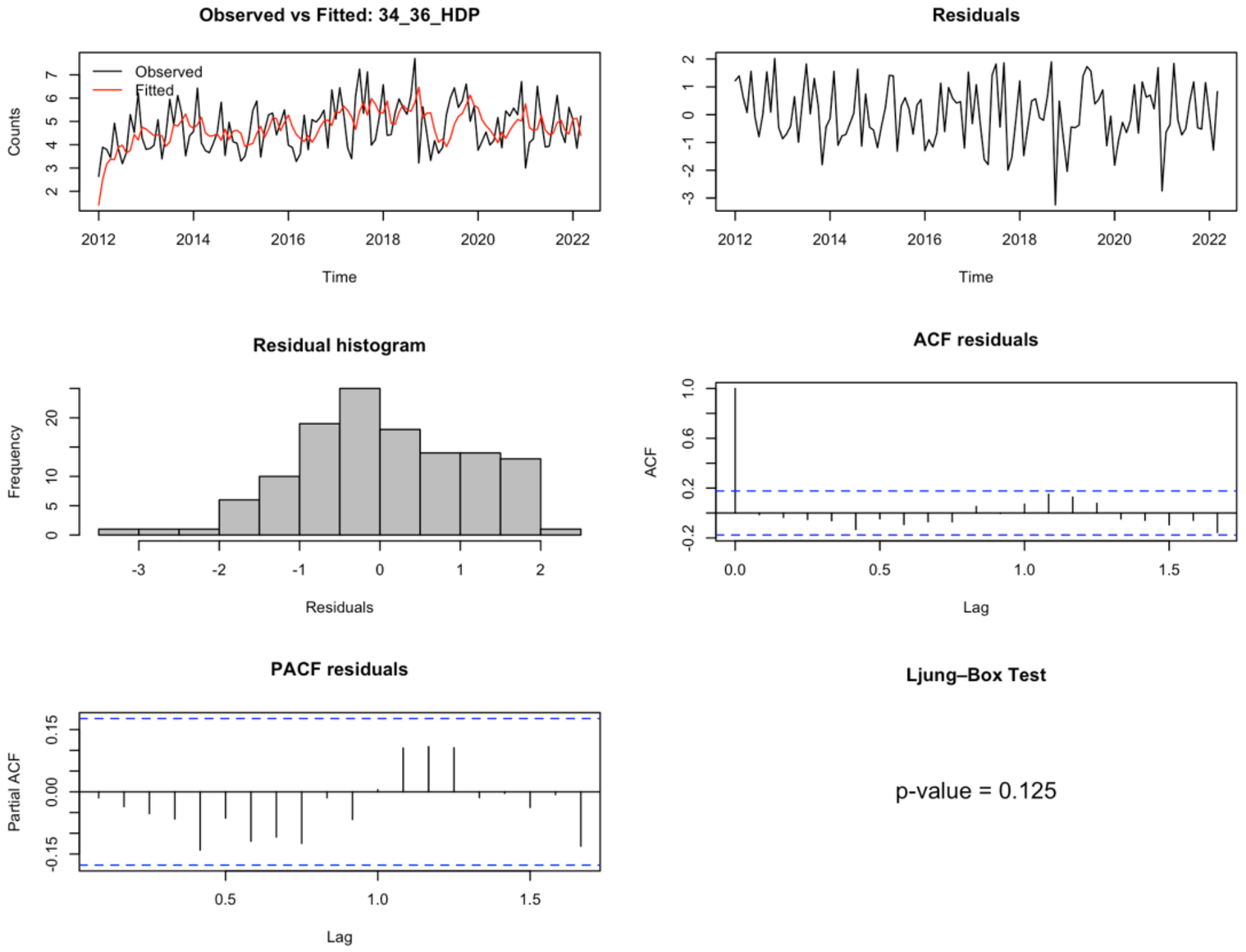

L. 34-36 Weeks GA without HDP

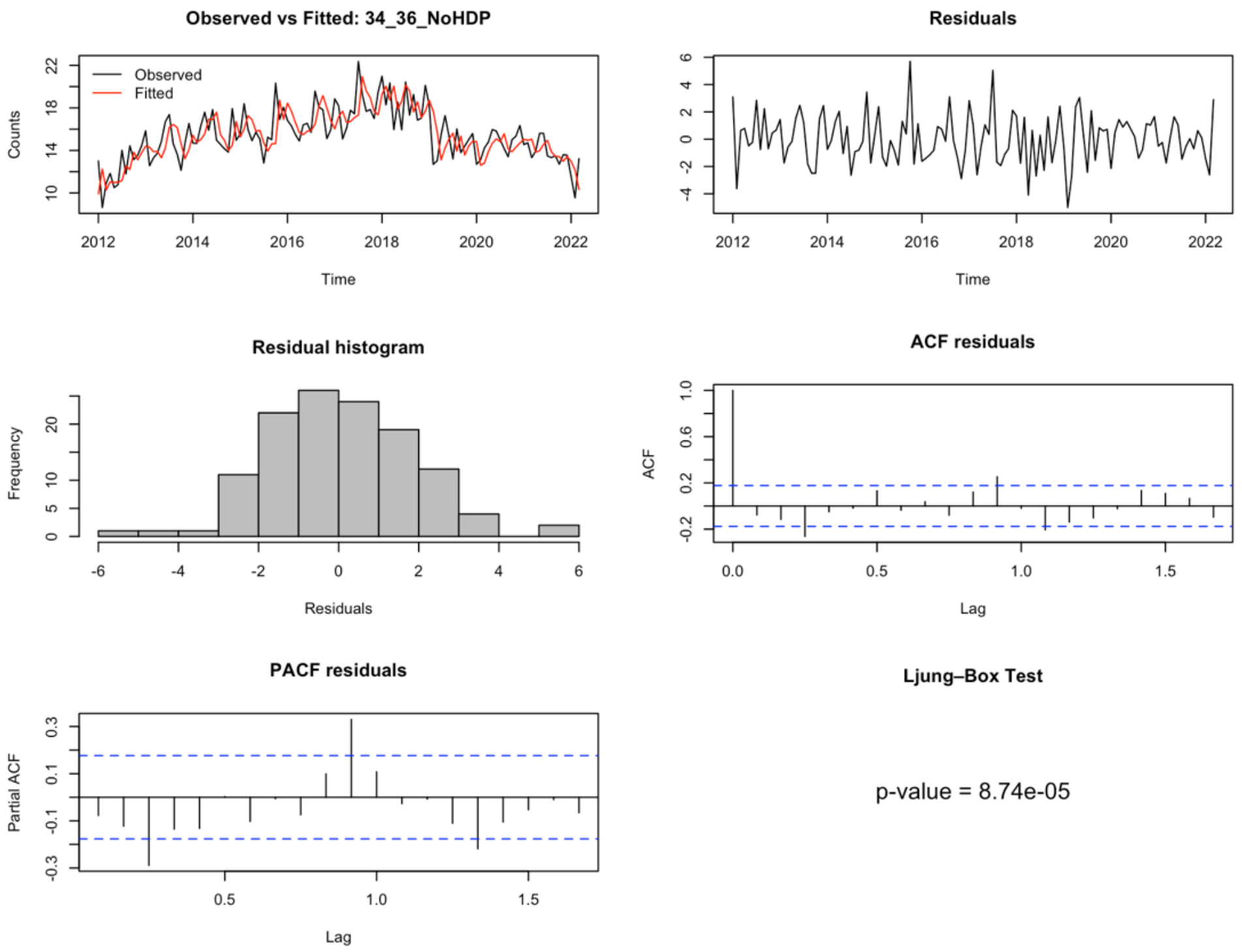

M. Private Hospital PTB with HDP

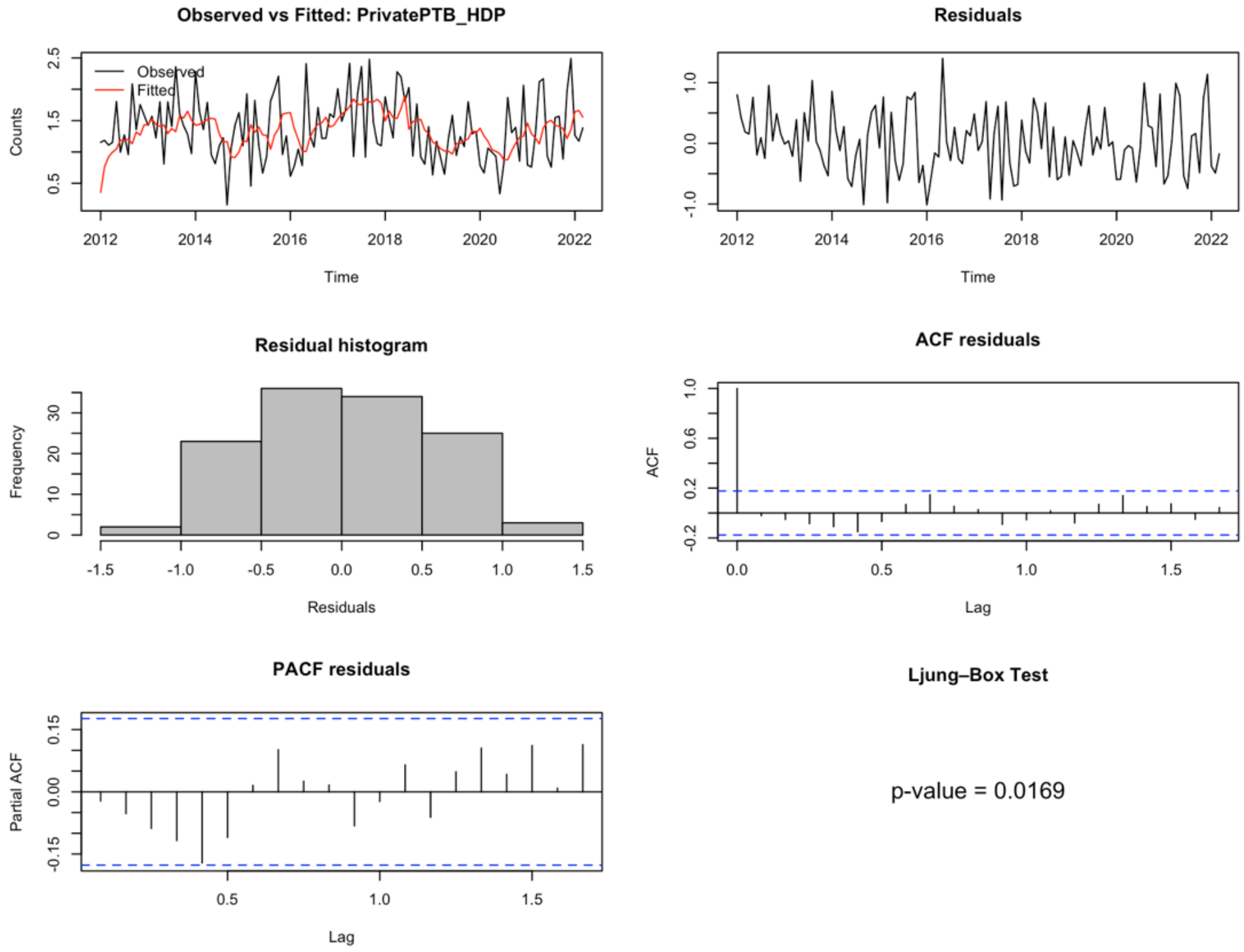

N. Private Hospital PTB without HDP

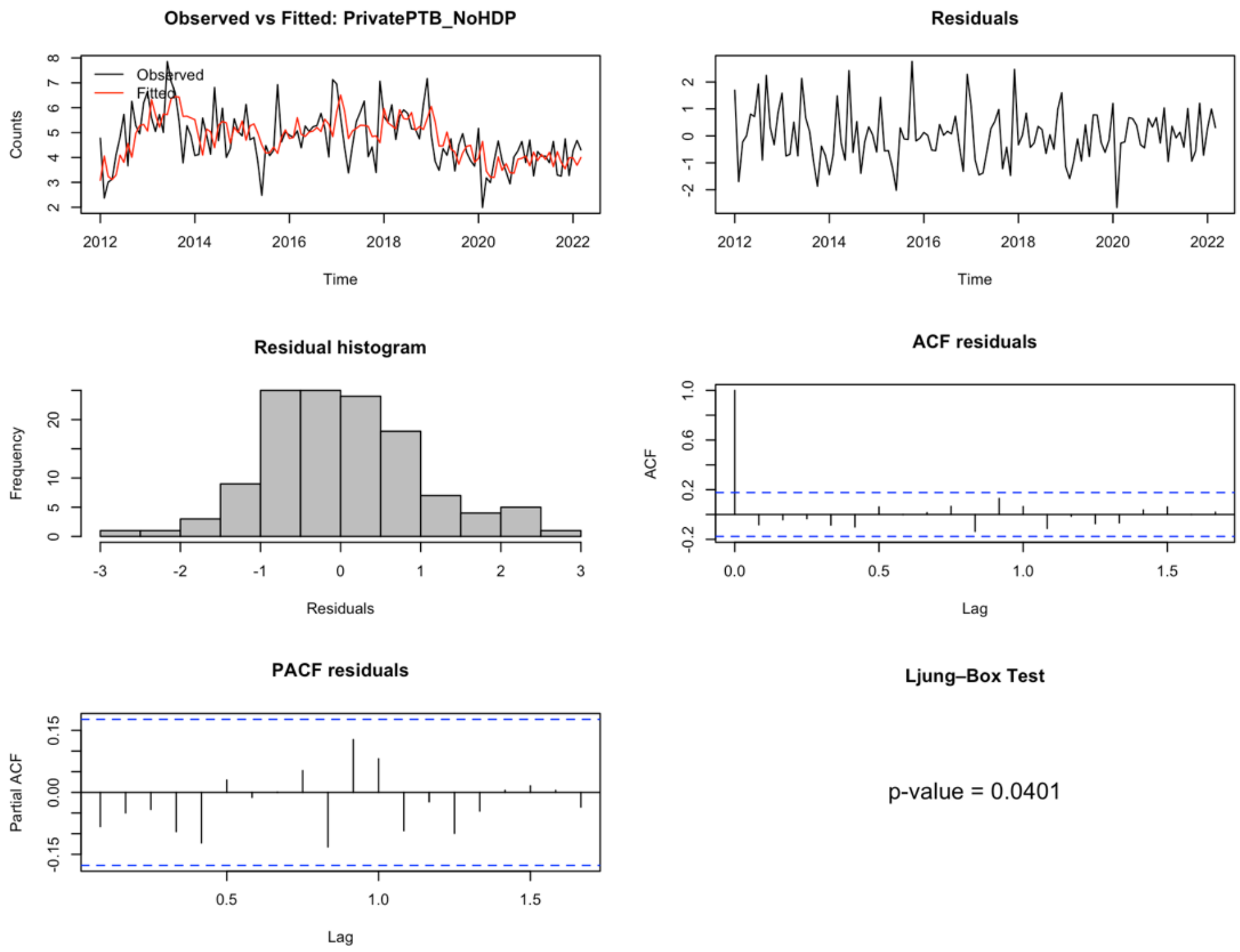

O. Public Hospital PTB with HDP

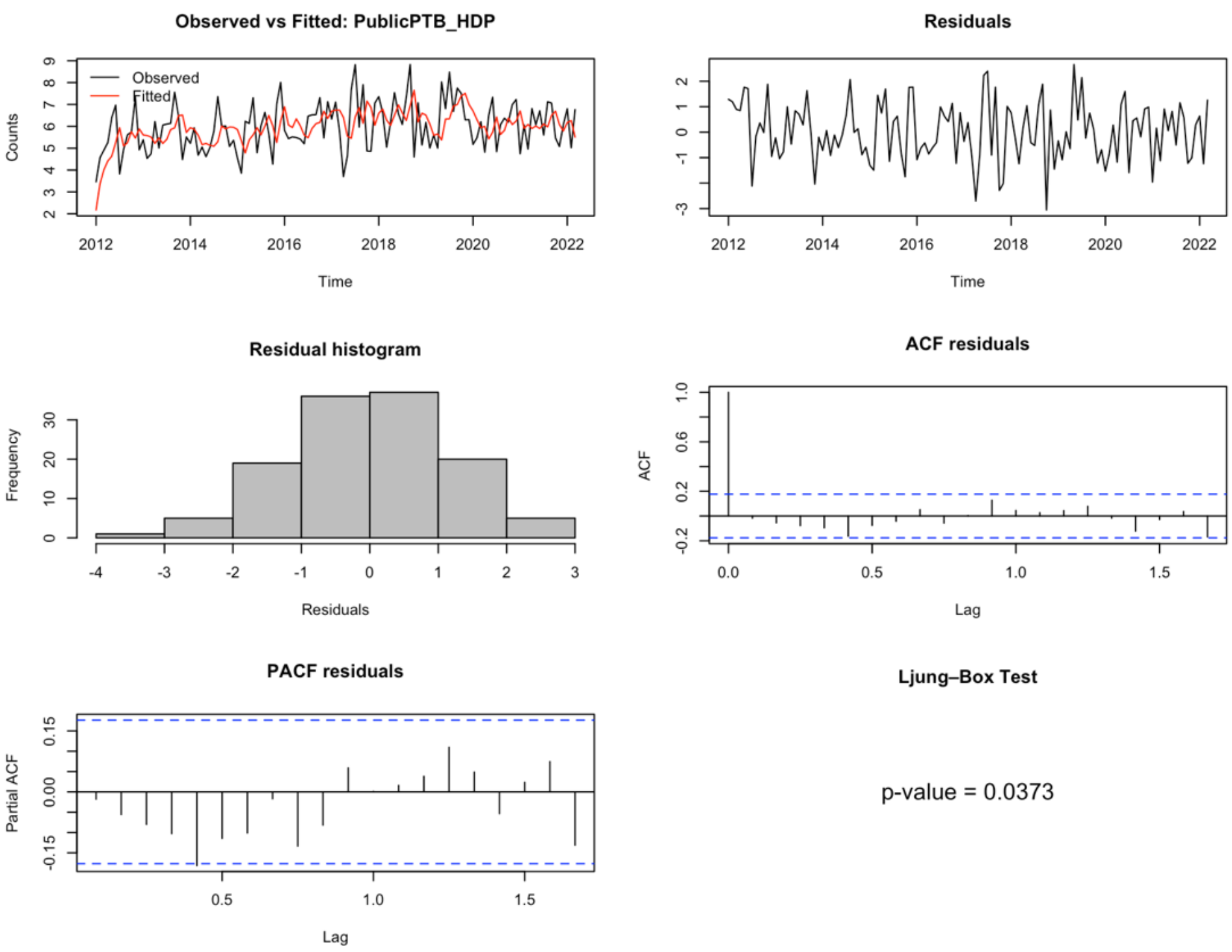

P. Public Hospital PTB without HDP

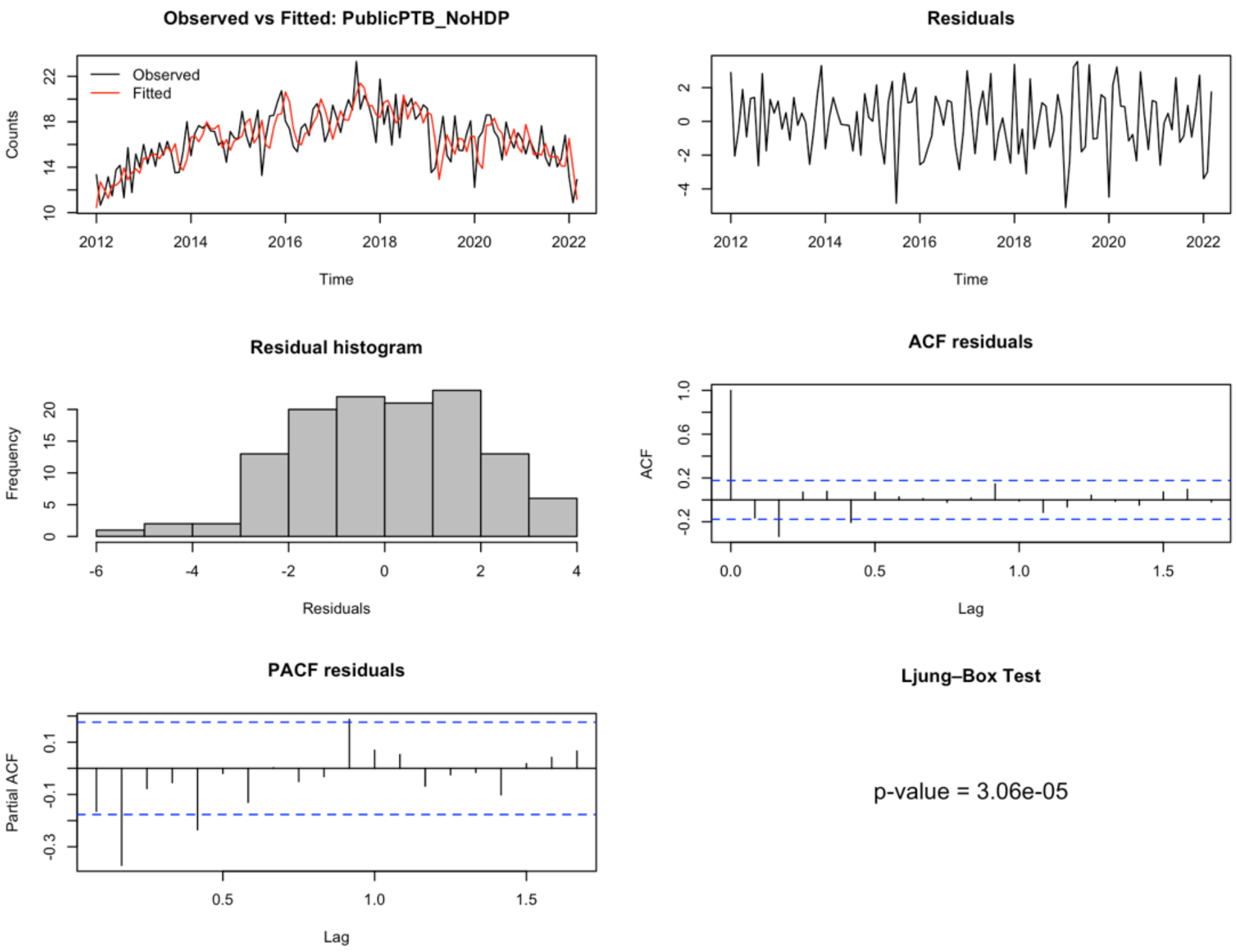

Q. Metropolitan Hospital with PTB with HDP

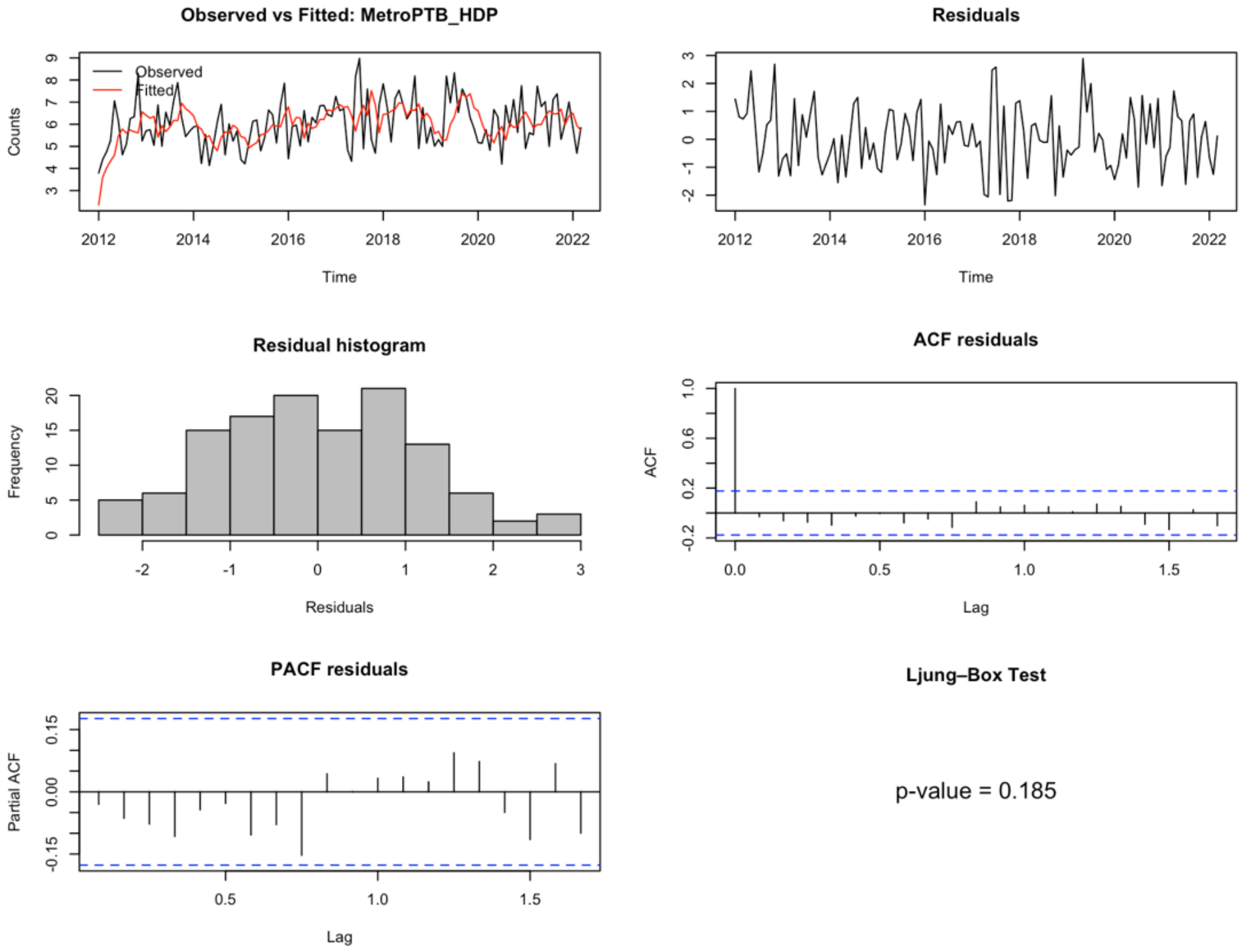

R. Metropolitan Hospital PTB without HDP

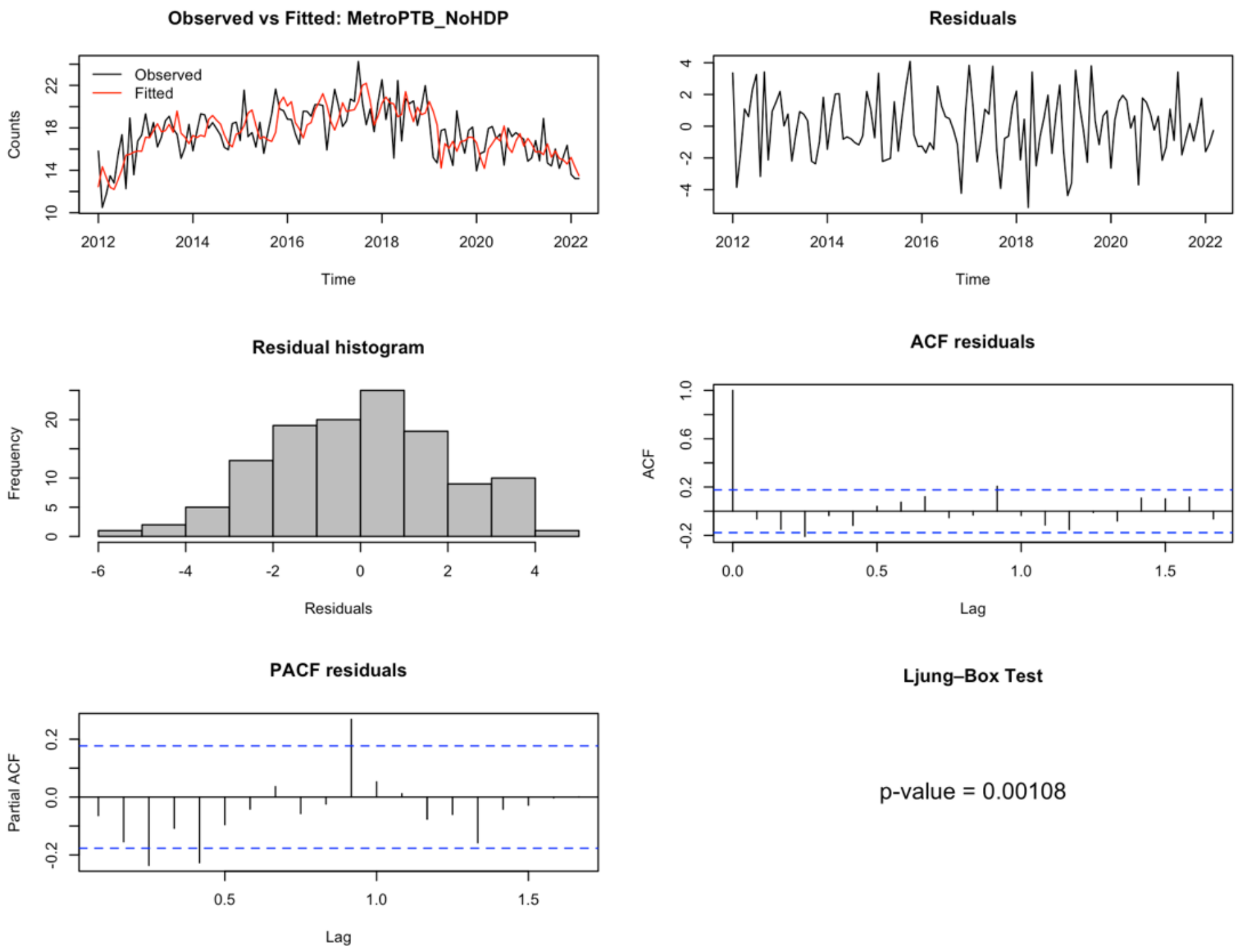

S. Regional Hospital PTB with HDP

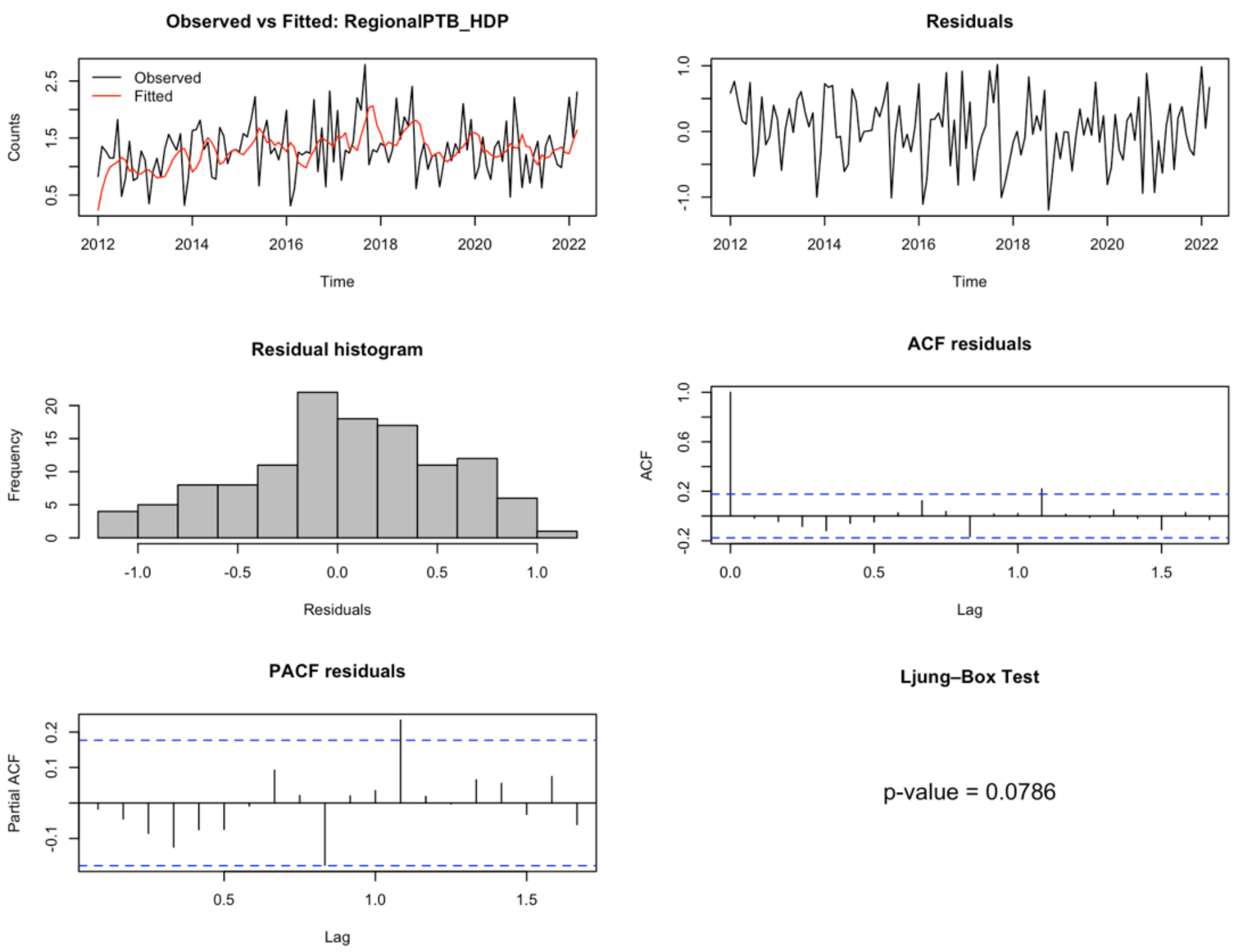

T. Regional Hospital PTB without HDP

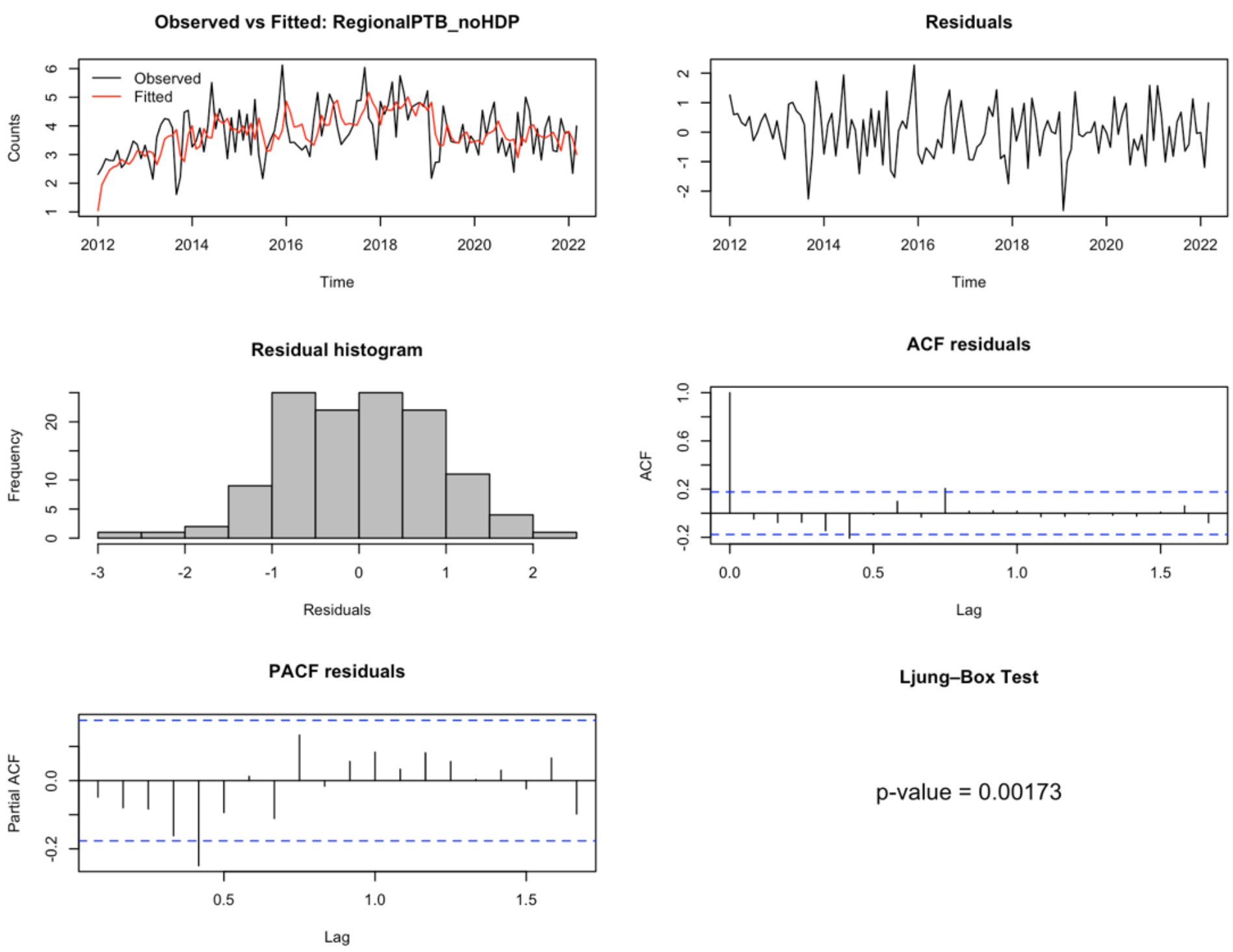

U. Low SEIFA PTB with HDP

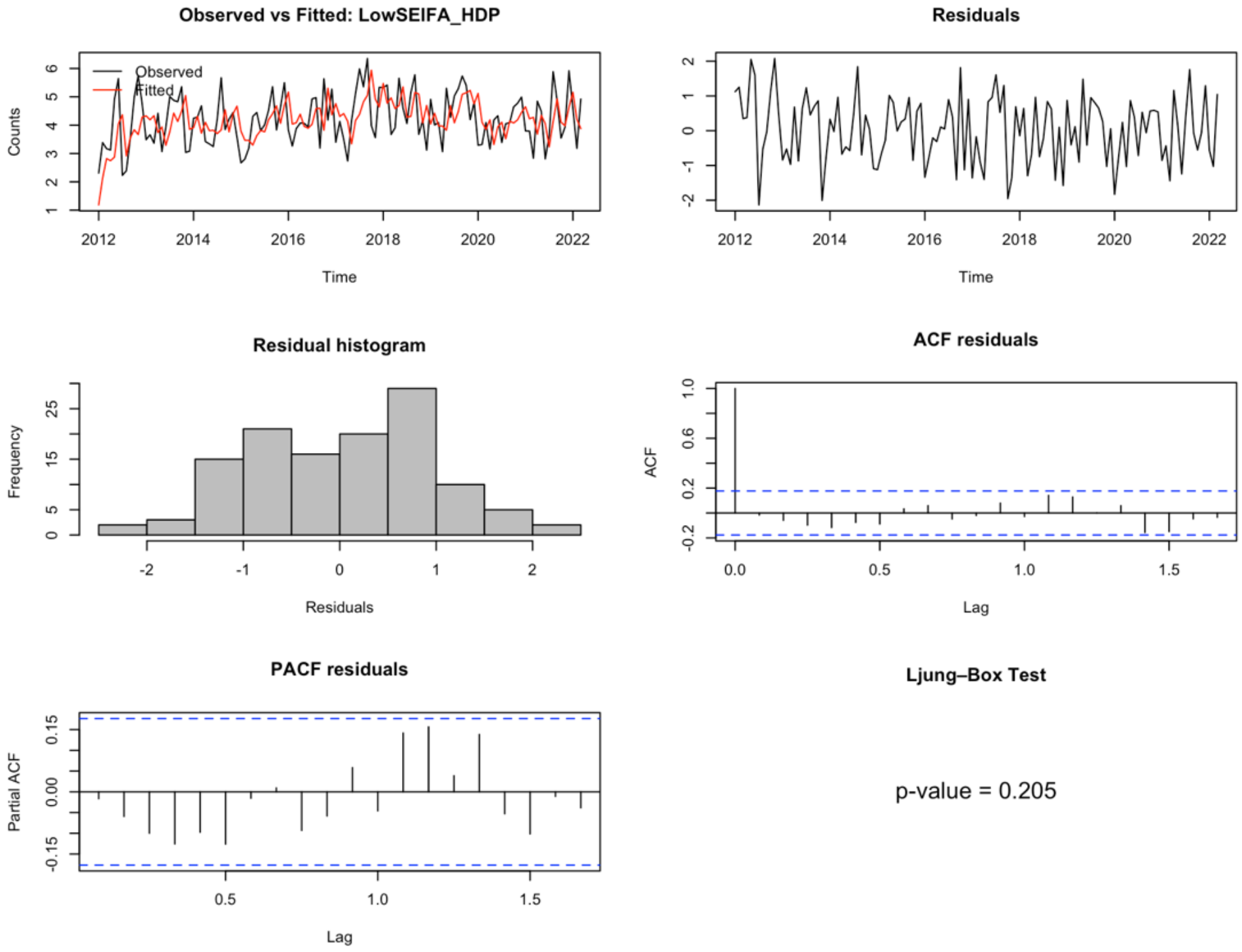

V. Low SEIFA PTB without HDP

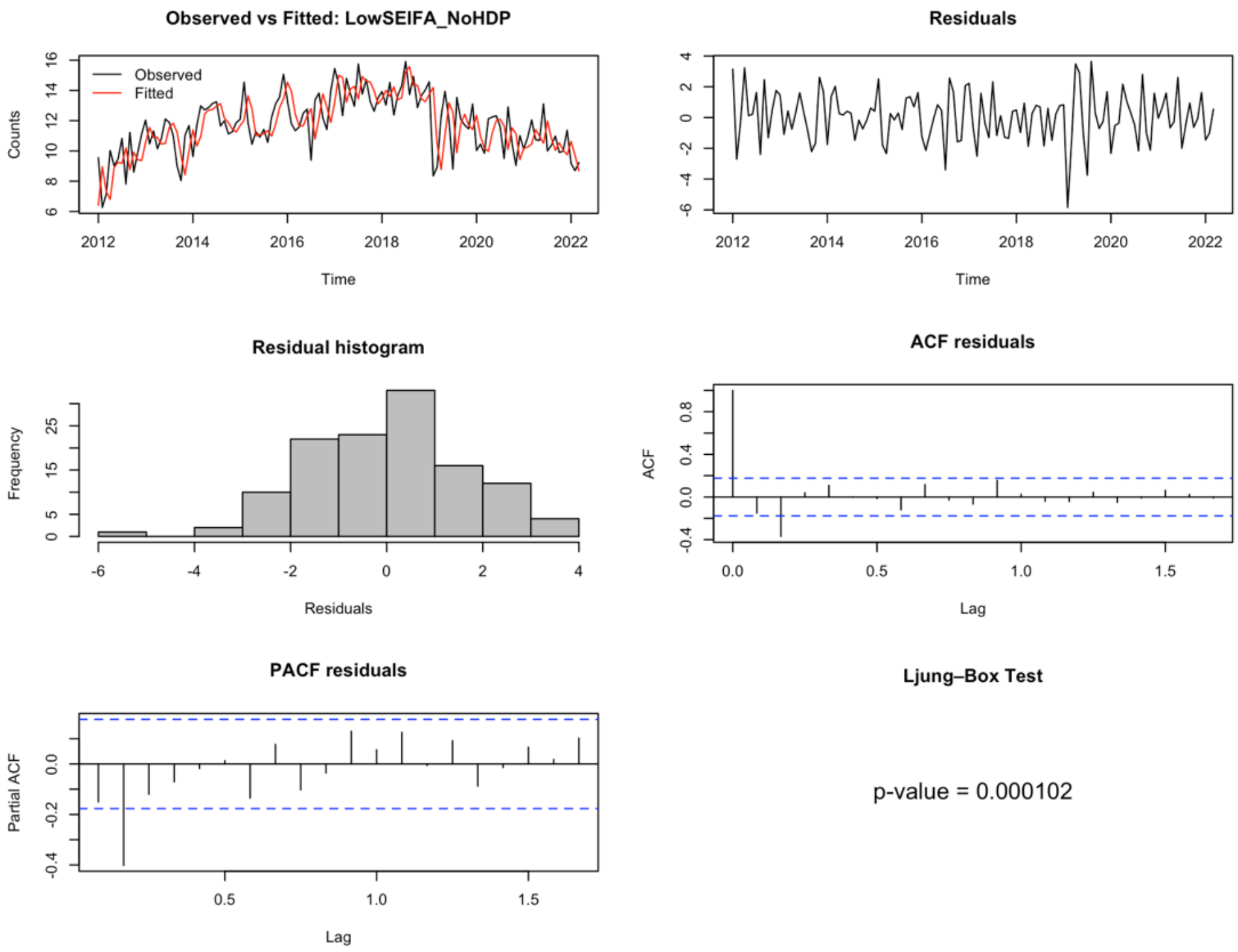

W. High SEIFA PTB with HDP

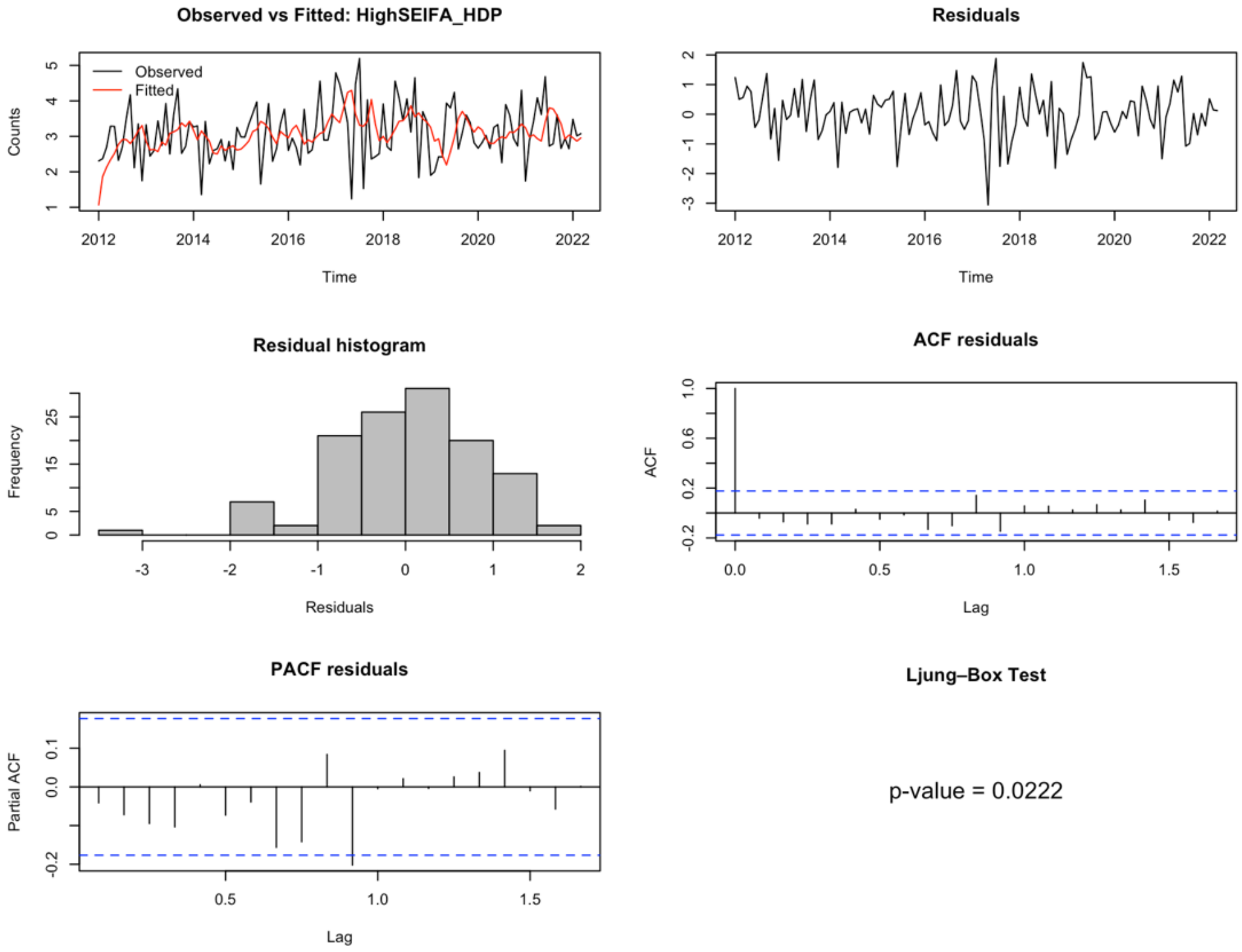

X. High SEIFA PTB without HDP

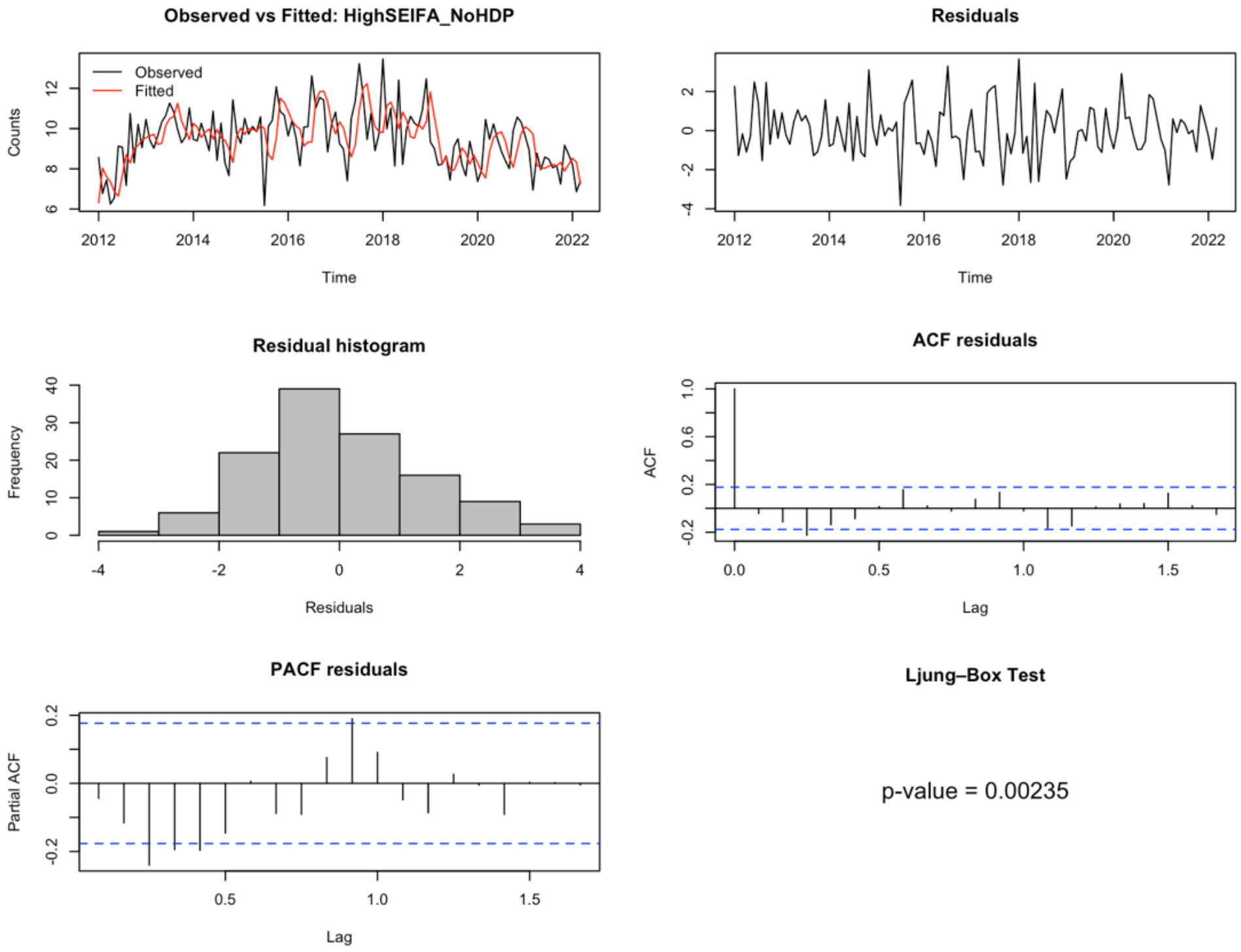

Box S2. Period-level decomposition analysis showing derivation of prevented cases

Calculation of expected PTB with HDP:

*Expected = Period 2 HDP cases × Period 1 PTB|HDP rate
= 23,157 × 0.1389
= 3,217 cases

Calculation of prevented cases:

†Offset = Expected - Observed
= 3,217 - 3,128
= 89 fewer cases than expected

Abbreviations: HDP = hypertensive disorders of pregnancy; PTB = preterm birth.

Despite HDP prevalence increasing by 0.66 percentage points (from 6.36% to 7.02%), the rate of PTB among women with HDP declined by 0.38 percentage points (from 13.89% to 13.51%). If the Period 1 rate had remained constant, we would expect 3,217 PTB with HDP cases during the Period 2. Instead, we observed 3,128 cases, representing 89 fewer cases than expected.

Note: This period-level decomposition differs from the year-by-year decomposition in Table S2, which uses 2012 as a fixed baseline rather than the Period 1 average

Table S6. Sensitivity analysis of seasonal autoregressive integrated moving average (SARIMA) analysis of preterm birth and hypertensive disorders of pregnancy trends by study period excluding COVID-19 lockdown periods (April 2020-onwards)

Primary and secondary outcomes	Period 1 Slope (95% CI) Jan 2012 – Jun 2018	Change in Slope (95% CI)	Period 2 Slope (95% CI) Jul 2018 – Mar 2022[a]
**PTB with HDP**	0.420 (-0.508, 1.348)	-0.257 (-2.426, 1.912)	0.163 (-1.622, 1.948)
p-value	0.37479	0.816337	0.85777
Iatrogenic PTB with HDP	0.091 (-0.020, 0.202)	-0.184 (-0.512, 0.143)	-0.093 (-0.372, 0.186)
p-value	0.10893	0.27052	0.51359
Spontaneous PTB with HDP	0.228 (-0.090, 0.546)	-0.440 (-1.222, 0.342)	-0.440 (-1.222, 0.342)
p-value	0.15936	0.2702449	0.5262569
**Total PTB**	0.445 (-0.700, 1.590)	-0.101 (-2.710, 2.508)	0.344 (-1.837, 2.525)
p-value	0.44618	0.9396167	0.7571499
Total iatrogenic PTB	0.313 (-0.121, 0.748)	-0.460 (-1.607, 0.687)	-0.147 (-1.106, 0.812)
p-value	0.15747	0.4317219	0.7640818
Total spontaneous PTB	0.200 (-0.521, 0.921)	-0.017 (-1.933, 1.898)	0.183 (-1.401, 1.767)
p-value	0.58596	0.985788	0.8208866
**Total HDP**	0.422 (-0.473, 1.317)	-0.225 (-1.703, 1.254)	0.197 (-1.122, 1.517)
p-value	0.3551	0.7659698	0.7692526
Term pregnancies with HDP	0.021 (0.002, 0.040)*	-0.004 (-0.036, 0.028)	0.017 (-0.011, 0.045)
p-value	0.026638	0.82103787	0.2207975
Term pregnancies without HDP	0.130 (0.115, 0.144)***	-0.129 (-0.358, 0.099)	0.000 (-0.228, 0.228)
p-value	0.00000	0.2674767	0.9978831
**PTB without HDP**	0.093 (-0.060, 0.245)	-0.119 (-0.525, 0.287)	-0.026 (-0.373, 0.320)
p-value	0.23385	0.565063	0.8807943
Iatrogenic PTB without HDP	0.091 (-0.020, 0.202)	-0.184 (-0.512, 0.143)	-0.093 (-0.372, 0.186)
p-value	0.10893	0.2705176	0.5135904
Spontaneous PTB without HDP	0.137 (-0.254, 0.528)	0.073 (-0.868, 1.014)	0.210 (-0.562, 0.982)
p-value	0.49274	0.8791993	0.5941678

Abbreviations: CI = confidence interval; HDP = hypertensive disorders of pregnancy; PTB = preterm birth.

Coefficients represent absolute monthly change in rate per 1 000 births | *<0.05, **<0.01, ***<0.001

[a]Period 1 slope (β_t) represents the baseline trend. The Change in Slope (β_{ramp1}) represents the shift in trajectory following the intervention. The Period 2 Slope is the absolute trend for the second period, calculated as the linear combination of the baseline slope and the change: Period 2 Slope = $\beta_t + \beta_{ramp1}$.

References

1. Australian Preterm Birth Prevention Alliance. How to prevent preterm birth. https://pretermalliance.com.au/mothers-to-be/how-to-prevent-preterm-birth/ (viewed Aug 2026).

2. Cleveland RB, Cleveland WS, McRae JE and Terpenning I. STL: a seasonal-trend decomposition procedure based on loess. Journal of Official Statistics. 1990; 6(1): 3-73.

3. Muggeo VMR. Estimating regression models with unknown break-points. Statistics in Medicine. 2003; 22(19): 3055-71.
